# Changes in Oviductal Cells and Small Extracellular Vesicles miRNAs in Pregnant Cows

**DOI:** 10.3389/fvets.2021.639752

**Published:** 2021-03-04

**Authors:** Rosane Mazzarella, Natália Marins Bastos, Alessandra Bridi, Maite del Collado, Gabriella Mamede Andrade, Jorge Pinzon, Cibele Maria Prado, Luciano Andrade Silva, Flávio Vieira Meirelles, Guilherme Pugliesi, Felipe Perecin, Juliano Coelho da Silveira

**Affiliations:** ^1^Molecular Morphology and Development Laboratory, Department of Veterinary Medicine, College of Animal Sciences and Food Engineering, University of São Paulo, Pirassununga, Brazil; ^2^Graduate Department, Faculty of Veterinary Medicine and Animal Science, National University of Colombia, Bogotá, Colombia; ^3^Molecular Endocrinology Physiology Laboratory, Department of Animal Reproduction, School of Veterinary Medicine and Animal Science, University of São Paulo, São Paulo, Brazil

**Keywords:** small extracellular vesicles, miRNAs, oviductal cells, oviductal fluid, embryo maternal-communication, reproduction, bovine

## Abstract

Early embryonic development occurs in the oviduct, where an ideal microenvironment is provided by the epithelial cells and by the oviductal fluid produced by these cells. The oviductal fluid contains small extracellular vesicles (sEVs), which through their contents, including microRNAs (miRNAs), can ensure proper cell communication between the mother and the embryo. However, little is known about the modulation of miRNAs within oviductal epithelial cells (OECs) and sEVs from the oviductal fluid in pregnant cows. In this study, we evaluate the miRNAs profile in sEVs from the oviductal flushing (OF-sEVs) and OECs from pregnant cows compared to non-pregnant, at 120 h after ovulation induction. In OF-sEVs, eight miRNAs (bta-miR-126-5p, bta-miR-129, bta-miR-140, bta-miR-188, bta-miR-219, bta-miR-345-3p, bta-miR-4523, and bta-miR-760-3p) were up-regulated in pregnant and one miRNA (bta-miR-331-5p) was up-regulated in non-pregnant cows. In OECs, six miRNAs (bta-miR-133b, bta-miR-205, bta-miR-584, bta-miR-551a, bta-miR-1193, and bta-miR-1225-3p) were up-regulated in non-pregnant and none was up-regulated in pregnant cows. Our results suggest that embryonic maternal communication mediated by sEVs initiates in the oviduct, and the passage of gametes and the embryo presence modulate miRNAs contents of sEVs and OECs. Furthermore, we demonstrated the transcriptional levels modulation of selected genes in OECs in pregnant cows. Therefore, the embryonic-maternal crosstalk potentially begins during early embryonic development in the oviduct through the modulation of miRNAs in OECs and sEVs in pregnant cows.

## Introduction

The oviduct provides the ideal microenvironment for early embryonic development (1, 2). The bovine embryo develops in the oviduct for 4 days, staying in contact with oviductal epithelial cells (OECs) as well as its secretions, the oviductal fluid (3). The oviductal epithelium is composed of ciliated cells and secretory cells that produce the oviductal fluid (4). The oviductal fluid is composed of simple and complex carbohydrates, proteins, ions, inorganic salts, lipids, phospholipids, small metabolites, and vesicles (4, 5). Changes in this fluid and the oviductal cells are regulated by different factors, such as the oviduct region (ampulla or isthmus), the ipsilateral or contralateral side to the corpus luteum (6–8), and the concentration of steroid hormones (9, 10).

The oviductal fluid and OECs are also affected by the presence of gametes and embryos. Different studies have demonstrated changes in gene expression patterns of bovine OECs co-cultured with embryos (11, 12). *In vivo*, despite no differences in OECs gene expression were found using microarray analysis in the presence of a single embryo in artificially inseminated heifers compared to cyclic heifers by Maillo et al. (13) or using target genes analysis by Rodríguez-Alonso et al. (14), the same group identified differentially expressed genes by RNA-seq when multiples embryos were transferred (13). In the oviductal fluid, different proteins were described indicating that the fluid composition could be influenced by the presence of gametes (15). Recently, Rodríguez-Alonso et al. (16) demonstrated that the oviductal fluid presents a different composition of carbohydrates, proteins, and amino acids in pregnant cows (artificially inseminated) when compared to cyclic cows (non-breed). Thus, it is possible that other components within the oviductal environment, such as extracellular vesicles present in the oviductal fluid, can also be modulated in pregnant animals.

EVs are nanoparticles secreted by cells in the extracellular environment (17) and contain bioactive materials, such as proteins (18), lipids (19), mRNAs, and non-coding RNAs, including microRNAs (miRNAs) (20, 21). EVs are classified as exosomes, microvesicles, and apoptotic bodies according to their size, biogenesis, and secretion (22). However, due to the difficulty to establish the specific origin of these EVs, they can be classified according to their size as small EVs (sEVs; <200 nm) or large EVs (lEVs; >200 nm) (23). In the oviduct, EVs have been identified in the oviductal fluid and are potentially related to the maternal crosstalk with gametes and embryos (24–28). Functionally, both sEVs originating from the oviductal fluid and bovine OECs conditioned culture medium are internalized by bovine embryos and improve *in vitro* embryo development and quality (29–31). However, the protein profile of sEVs that originated from OECs *in vivo* is different from the sEVs produced by OECs *in vitro* (31). Lopera-Vasquez et al. (29) demonstrated that sEVs from the isthmus have a better effect on *in vitro* embryo quality than sEVs from the ampulla. Although they have not studied the content of these sEVs, it can be inferred that the contents from the isthmus are somehow different and better for the embryo. The molecular contents of oviductal sEVs (RNA, protein, and small ncRNA, including miRNAs) are also regulated by steroid hormones across the bovine estrous cycle (32). To our knowledge, there are no studies on how the sEVs miRNA contents are modulated in pregnant cows.

miRNAs are small non-coding molecules (~22 nucleotides) that act in post-transcriptional regulation (33, 34). miRNAs are transcribed as primary miRNAs (pri-miRNAs), which are processed in precursor miRNA (pre-miRNA) and then transferred to the cytoplasm for further processing into mature miRNAs sequences (35). In mammals, the partial complementary base pairing miRNA-mRNA can induce transcription inhibition, thus regulating protein levels (36). miRNAs are involved in different reproductive events, such as spermatogenesis (37), follicular development (38, 39), oocyte maturation (40, 41), and embryo development (42). To our knowledge, there are no studies on miRNAs regulation and function in OECs from pregnant cows. miRNAs are also involved in cell-to-cell communication through sEVs (43). miRNAs within sEVs may play a role in modulating embryo-maternal communication during early pregnancy (44, 45). EVs can mediate the delivery of its miRNAs contents into embryos, a phenomenon already implicated in embryo development and maternal-embryonic communication (45–48). However, there is no evidence of the effects on miRNA contents of the oviductal sEVs from pregnant cows during the first embryo cleavages at the oviduct.

Therefore, the modulation and function of miRNAs within oviductal epithelial cells and EVs from the oviductal fluid in early pregnant cows is still elusive. Thus, we hypothesize that the miRNAs contents in small extracellular vesicles from the oviductal flushing (OF-sEVs) and in oviductal epithelial cells (OECs) undergo differential modulations in pregnant cows. The present study evaluated the profile of 383 miRNAs in OF-sEVs and OECs in artificially inseminated (pregnant group) and in sham-inseminated (non-pregnant group) cows. Additionally, we access in OECs genes known to influence the immune system, inflammation, and early pregnancy development. To our knowledge, this is the first study to characterize the changes in miRNA profile from both OF-sEVs and OECs obtained from pregnant and non-pregnant cows. Additionally, functional enrichment analysis was performed to evaluate the predicted pathways related to differentially expressed miRNAs between pregnant and non-pregnant cows.

## Materials and Methods

All the reagents were purchased from Sigma–Aldrich Chemical Company (St. Louis, MO) unless otherwise stated. The present study was approved by the University of São Paulo Research Ethics Committee (protocol number: 4909010817).

### Animal Model

This study was conducted at the University of São Paulo (campus of Pirassununga, SP, Brazil) with Nelore (*Bos indicus*) multiparous cows with a body condition score 4 (0-emaciated; 5-obese) according to Ayres et al. (49). Cows were fed with corn silage, supplemented with mineral salt, and free access to potable water. In order to obtain the oviducts exposed or not exposed to embryos, 25 cows were submitted to estrous synchronization protocol. As described by Gonella-Diaza (50) with minor modifications: on the first day, all the cows received 2 mg of estradiol benzoate (2 ml, i.m., Sincrodiol, Ourofino Saúde Animal, Brazil), 0.53 mg of sodium cloprostenol (2 ml, i.m., Sincrocio, Ourofino Saúde Animal, Brazil) and an intravaginal P4-releasing device (1 g; Sincrogest®, Ourofino Saúde Animal, Brazil). On day 7, the P4 device was removed, and cows received 0.53 mg of sodium cloprostenol (2 ml, i.m., Sincrocio, Ourofino Saúde Animal, Brazil). On day 9, cows received 0.01 mg of gonadotrophin releasing hormone (GnRH) analog (2.5 ml, i.m., Sincroforte, Ourofino Saúde Animal, Brazil). Twelve hours after GnRH analog application, cows were randomly assigned into two groups: fixed-time artificial inseminated (FTAI, *n* = 14) or sham-inseminated (control, *n* = 11). In the group FTAI, cows were artificially inseminated with frozen-thawed semen from the same bull of proved fertility. In the control group, cows were sham-inseminated with deposition of depleted semen extender in the uterine tract. For sperm depletion, straws from the same bull used in the FTAI group were thawed, centrifuged at 4,000 × g for 15 min, and the supernatant was filled into a new straw. The ovulation time was controlled by ultrasound (MyLab Delta, Esaote, Italy) and only animals that had an ovulated follicle between 24 and 36 h after GnRH analog application were kept in this study. All cows were slaughtered about 120 h after ovulation induction with GnRH at the slaughterhouse at University of São Paulo (campus of Pirassununga/SP, Brazil).

### Sample Collection and Groups Formation

Reproductive tracts were processed within 30 min after slaughter. Oviducts ipsilateral to the corpus luteum (CL) were dissected and separated from the utero-tubal junction. Next, oviducts were divided in ampulla and isthmus through the ampullary–isthmic junction, identified as the place where the oviductal diameter starts to decrease, as described by Maillo et al. (51) and Gonella-Diaza et al. (52). The isthmus was individually flushed with 1 ml of calcium and magnesium-free PBS with 0.1% of polyvinylpyrrolidone (PVP). The presence or absence of the embryo within the isthmus portion was used for group formation and confirmed in the oviductal flushing using a stereo microscope. After washing, epithelial cells of each isthmus were obtained by squeezing the tissue using a sterile glass slide and frozen in liquid nitrogen until further use.

In addition to the criterion of the presence or absence of the embryo, we also analyzed pre-ovulatory follicle (POF) diameter, weight, and diameter of the CL and serum concentration of progesterone (P4) and estrogen (E2). The POF diameter was measured by ultrasound (MyLab Delta, Esaote, Italy) on day 9 of the estrous synchronization protocol. For CL weight and size, CLs were dissected from the ovary, weighed, and their diameter was calculated as the average between two perpendicular axes measured. For serum concentration of P4 and E2, blood samples were collected from the jugular vein in serum blood collection tubes (BD, São Paulo/SP, Brazil) at the time of slaughter. The serum was separated by centrifugation at 1,500 × g for 30 min, stored at −20°C, and the measurement of E2 and P4 was performed using chemiluminescence immunoassay (ADVIA Centaur®-Siemens) at Pasin Laboratory (Santa Maria/RS, Brazil).

### Isolation of OF-sEVs

OF-sEVs were obtained from the isthmus of ipsilateral oviducts from pregnant and non-pregnant groups according to the isolation protocol previously used by da Silveira et al. (53), Alminaña et al. (32), and Ávila et al. (54). Briefly, the oviductal flushing was centrifuged at 300 × g for 10 min to remove live cells, 2,000 × g for 10 min to remove cellular debris, 16,500 × g for 30 min to remove large vesicles, and the remaining supernatant was placed at −80°C until further use. The supernatant was filtered through a 0.20 μm sterile syringe filter (PES membrane; Corning) in order to remove any remaining large EVs. For sEVs isolation, filtered oviductal flushing was centrifuged twice at 119,700 × g for 70 min at 4°C (Optima XE-90 Ultracentrifuge; rotor 70 Ti; Beckman Coulter). After this procedure, the supernatant was discarded, and the sEVs pellets were resuspended in 20 μL of phosphate-buffered saline (1 × Ca^2+^/Mg^2+^ free PBS; 137 mM NaCl, 2.7 mM KCl, 10 mM Na_2_HPO_4_, 2 mM KH_2_PO_4_) and used for further analysis. Due to the small volume of oviductal flushing obtained from a single animal, the protocol to isolate OF-sEVs was validated using female reproductive tracts obtained from slaughterhouses, as described in Supplementary Data 1.

### Nanoparticle Tracking Analysis

The OF-sEVs isolated from pregnant and non-pregnant groups were analyzed based on particle mode size and concentration by nanoparticle tracking analysis (NTA). OF-sEVs isolated from 200 μL of each oviductal flushing were individually centrifuged, and their pellets were resuspended in 20 μL of 1 × Ca^2+^/Mg^2+^ free PBS. As described by Ávila et al. (54), with minimal modifications, particle size, and concentration were measured by nanoparticle tracking analysis using Nanosight (NS300; Malvern). Briefly, for this analysis, the dilution factor used was 1: 100 in 1 × Ca^2+^/Mg^2+^ free PBS. Five videos of 30 s were taken for each sample, captured by a scientific complementary metal–oxide–semiconductor (sCMOS) camera at camera level 13 and under a controlled temperature of 38.5°C. Considering the threshold level of 5, these captured images were tracked by the NanoSight NTA 3.4 Analytical Software (NTA 3.4 Build 3.4.003; Malvern) that measures and provides information about particle size and concentrations of each sample.

### Total RNA Extraction, Reverse Transcription, and Real-Time PCR

Total RNA, including miRNAs, from OF-sEVs and OECs was extracted according to the TRIzol reagent (Thermo Fisher Scientific) manufacturer's instruction and with the addition of 1.33 μL the coprecipitator GlycoBlue (Thermo Fisher Scientific) to the aqueous phase before RNA precipitation, as previously described by Da Silveira et al. (55) and Gonella-Diaza et al. (56) with minimal modifications. Total RNA concentrations were analyzed using spectrometry (NanoDrop 2000, Thermo Fisher Scientific). The RNA was treated with DNaseI (Invitrogen; Carlsbad, CA) according to the manufacturer's instructions.

miRNA analysis of OF-sEVs and OECs were performed as described in Da Silveira et al. (55). Briefly, for cDNA synthesis, reverse transcription was performed with the commercial miSCRIPT II RT kit (QIAGEN), using the MiScript HiFlex Buffer and 200 ng of total RNA. For quantitative RT-PCR, the miScript SYBR® Green PCR Kit (QIAGEN) was used according to the manufacturer's instructions. The relative levels of 383 miRNAs were evaluated for each sample. Three hundred eighty four-well plates were assembled using a multichannel electronic pipetting system and analyzed by RT-PCR with QuantStudio 6 Flex (Applied Biosystems). miRNAs were considered present when cycle threshold (CT) was <37 in at least three out of six biological repetitions with an adequate melting curve. For both OF-sEVs and OECs, the CT values were normalized using the geometric mean CT of the endogenous genes Hm/Ms/Rt U1 snRNA and bta-miR-99b (57). The relative expression values were calculated using the ΔCt method, and the normalized data were transformed by 2^−ΔCt^ for graphical representation of the relative transcript levels (58).

Regarding mRNAs analysis of OECs, we evaluated the relative expression of 14 mRNAs selected from key pathways known to influence oviductal function and two endogenous controls (Table 1) for each sample. Briefly, reverse transcription reaction was performed with 30 ng of total RNA per gene using High-Capacity cDNA Reverse Transcription Kit (Thermo Fisher Scientific), according to the manufacturer's protocol. For quantitative RT-PCR, the GoTaq® qPCR Master Mix (Promega) was used according to the manufacturer's instructions and using the QuantStudio 6 Flex (Applied Biosystems) with the following PCR cycle conditions: 95°C for 10 min, 45 cycles of 95°C for 15 s, and 60°C for 60 s. The amplification of single cDNA products was confirmed by melt curve analysis. CT values were normalized using the geometric mean CT of the endogenous genes PPIA and GAPDH, as previously described for similar tissues (50). The relative expression values were calculated using the ΔCt method, and the normalized data were transformed by 2^−ΔCt^ for graphical representation of the relative transcript levels (58).

**Table 1:** Primer sequences of genes analyzed by real-time PCR.

**Key function**	**Gene symbol**	**Gene name**	**Primer sequences (5^**′**^-3^**′**^)**	**GenBank number**	**References**
Inflammatory and immune response	*IL1B*	Interleukin 1 Beta	F: TCCAGCCAACCTTCATTGC R: ACAGCTCATTCTCGTCACTGTAGTAAG	NM_174093.1	LFEM^*^
	*IL2RA*	Interleukin 2 Receptor Subunit Alpha	F: CTGCTCATATCCCCCAAAGG R: CATGGGTTTGCTTGCTGTCTT	NM_174358.2	LFEM^*^
	*IL6R*	Interleukin 6 Receptor	F: ACCACCAAGGCCGTGTTACT R: GGCGACACACAGGGACAATA	NM_001110785.1	(59)
	*TAB1*	TGF-Beta-Activated Kinase 1-Binding Protein 1	F: GACGCGCTGGCTGAGAAG R: GCCTTGAGTCTTTCGAGGATCTT	NM_001102057.1	(59)
	*TGFB1*	Transforming Growth Factor Beta 1	F: CCTGCTGAGGCTCAAGTTAAAAG R: GCCACTGCCGCACAACTC	NM_001166068.1	(60)
	*TNFR1*	Tumor Necrosis Factor Receptor Type 1	F: AGGACCCAGGCACTACAGTACTATTAC R: CACCGCTGGTAGCGACATG	NM_174674.1	(59)
	*RGS2*	Regulator Of G Protein Signaling 2	F: CCTGAACACCCAGGCCTGAAT R: GAACCCCATGCCACATGAGA	NM_001075596.1	LFEM^*^
	*PTGES2*	Prostaglandin E synthase 2	F: GTGGGCGGACGACTGGTTGG R: CGGAGGTGGTGCCTGCGTTT	NM_001166554.1	(61)
	*PTGES3*	Prostaglandin E synthase 3	F: CAGTCATGGCCAAGGTTAACAAA R: ATCACCACCCATGTTGTTCATC	NM_001007806.2	(61)
Pregnancy recognition	*IFNAR2*	Interferon (alpha, beta and omega) receptor 2	F: CTGGTCATTTGTATGGGCTCTTT R: GTATCCCGGGACTGTCGAATT	NM_174553.2	(62)
	*MX1*	MX dynamin-like GTPase 1	F: GTACGAGCCGAGTTCTCCAA R: ATGTCCACAGCAGGCTCTTC	NM_173940.2	(63)
	*MX2*	MX dynamin-like GTPase 2	F: CTTCAGAGACGCCTCAGTCG R: TGAAGCAGCCAGGAATAGT	NM_173941	(63)
	*OAS1Y*	2',5'-oligoadenylate synthetase 1	F: GCGGACCCTACAGGAAATGT R: TGTTCTTGGGGCGACACATC	NM_001040606.1	LFEM^*^
	*ISG15*	ISG15 ubiquitin-like modifier	F: AGAGAGCCTGGCACCAGAAC R: TTCTGGGCGATGAACTGCTT	NM_174366.1	(62)
Endogenous control	*PPIA*	Peptidylprolyl isomerase A	F: GCCATGGAGCGCTTTGG R: CCACAGTCAGCAATGGTGATCT	NM_178320.2	(64)
	*GAPDH*	Glyceraldehyde-3-phosphate dehydrogenase	F: GCCATCAATGACCCCTTCAT R: TGCCGTGGGTGGAATCA	NM_001034034.2	(64)

*Gene selected from key pathways known to influence oviductal function and endogenous controls. F, Forward and R, Reverse*.

* *Provided by Molecular Endocrinology Physiology Laboratory (LFEM-FMVZ-USP)*.

### Statistical and Bioinformatics Analysis

The data obtained were tested for outliers' presence, normality by Shapiro–Wilk test, and the normalized RT-PCR data were analyzed by the Student's *t*-test. For all analyzes, *p* < 0.05 was considered significant unless otherwise stated. Statistical analyzes were performed with JMP 14 (SAS Institute Inc.). For bioinformatics analysis and identification of biological pathways, the miRNAs differently detected within the groups were evaluated by miRWalk 2.0 bovine database (65). MiRWalk is an online database that, through their TarPmiR algorithm, allowed the searching of validated and predicted interactions between miRNAs and gene sequences. Then, the biological pathways predicted as modulated by these miRNAs of interest were identified through the gene set enrichment analysis (GSEA) function within the MiRWalk platform, assessing the geneset “KEGG Pathways.” Only pathways with a *p*-value lower than 0.05 were considered as significant.

## Results

### Sample Collection and Groups Formation

Embryos were found in six out of the 14 cows in FTAI group, and these samples formed the Pregnant group (*n* = 6). Six out of the 11 sham-inseminated cows that followed the analyzed variables described in Table 2 formed the Non-Pregnant group (*n* = 6). No differences were found on diameter POF size, weight, and diameter of the CL as well as serum concentration of P4 and E2 between the two groups, ensuring that these variables did not interfere with our results.

**Table 2:** Variables analyzed for group formation.

**Variables**	**Group**		***P*-value (*t*-test)**
	**Non-pregnant (*n* = 6)**	**Pregnant (*n* = 6)**	
Pre-ovulatory follicle (mm)	13.30 ± 1.19	13.08 ± 2.53	0.8546
Corpus luteum weight (g)	1.40 ± 0.27	1.36 ± 0.50	0.8492
Corpus luteum diameter (cm)	1.57 ± 0.08	1.45 ± 0.26	0.3330
Serum progesterone concentration (ng/mL)	3.96 ± 1.17	3.82 ± 1.90	0.8757
Serum estrogen concentration (pg/mL)	25.07 ± 2.32	26.52 ± 7.92	0.6824

*Values are expressed as means ± SEM*.

### Isolation of OF-sEVs

Initially, the efficiency of the sEVs isolation protocol was confirmed using OF-sEVs obtained as described in the Supplementary Data 1. TEM was used to analyze the morphology and size of the isolated OF-sEVs. Throughout the images, we were able to identify cup-shaped particles with characteristic sizes resembling sEVs (Supplementary Figure 1). Therefore, the isolation protocol was adequate for the isolation of OF-sEVs.

### Nanoparticle Tracking Analysis

**Figure 1:**
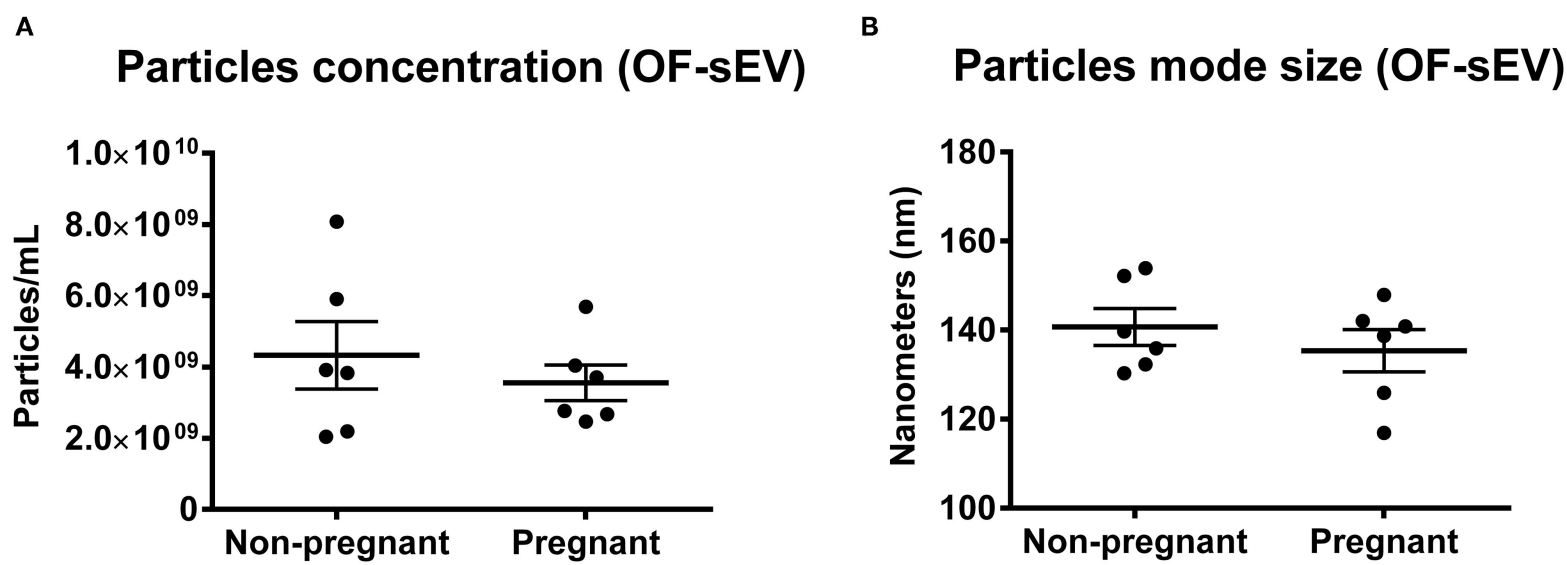
Characterization of OF-sEVs by nanoparticle analysis. Nanoparticle tracking analysis showing no difference in particles concentration **(A)** and mode size **(B)** between the groups OF-sEV/Non-pregnant (*n* = 6) and OF-sEV/Pregnant (*n* = 6). Error bars represents standard errors of the means.

In order to analyze particle concentration and mode size of sEVs, we performed NTA using OF-sEVs isolated from non-pregnant and pregnant cows. No differences were identified in mean particle concentration (OF-sEV/Non-pregnant: 4.3 × 10^9^ ± 2.3 × 10^9^ particles/mL; OF-sEV/Pregnant: 3.5 × 10^9^ ± 1.2 × 10^9^ particles/mL; Figure 1A) or mean mode size (OF-sEV/Non-pregnant: 140.7 ± 10.1 nm; OF-sEV/Pregnant: 135.4 ± 11.6 nm; Figure 1B) between the groups. From this result, we concluded that the pregnancy does not affect concentration and size of sEVs in the oviductal flushing.

### miRNA Profile of OF-sEVs and Bioinformatics Analyses

**Figure 2:**
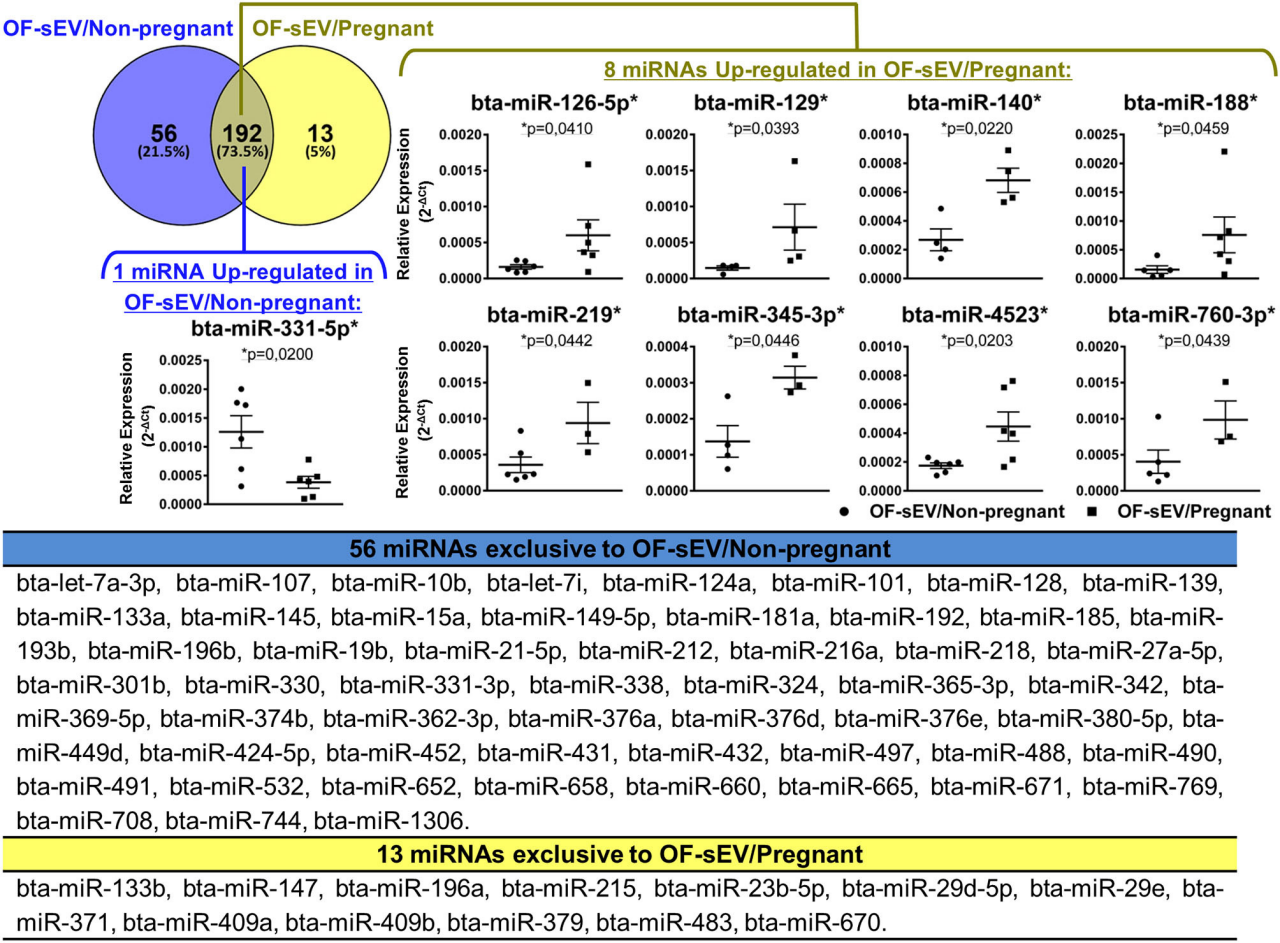
miRNAs profile of OF-sEVs isolated from non-pregnant and pregnant cows. Venn diagram representing the 261 miRNAs detected: 192 in common between the two groups, 56 exclusive to OF-sEV/Non-pregnant and 13 exclusive to OF-sEV/Pregnant. Among the 192 miRNAs detected in both groups, eight miRNAs (bta-miR-126-5p, bta-miR-129, bta-miR-140, bta-miR-188, bta-miR-219, bta-miR-345-3p, bta-miR-4523, and bta-miR-760-3p) were up-regulated in OF-sEV/Pregnant and one miRNA (bta-miR-331-5p) was up-regulated in OF-sEV/Non-pregnant. The asterisk (*) indicates miRNAs with relative expression significantly different (*p* < 0.05) within the groups. Samples size were *n* = 6 for both groups. Error bars represent SEM.

To analyze the miRNAs profile, the relative expression levels of 383 miRNAs were investigated in OF-sEVs from six oviducts from pregnant and six oviducts non-pregnant cows. The presence of 248 and 205 miRNAs were identified in OF-sEVs isolated from the non-pregnant and pregnant group, respectively. Of these, 192 were commonly detected between the two groups, 56 were exclusive to OF-sEV/Non-pregnant, and 13 were exclusive to OF-sEV/Pregnant (for CT levels, see Supplementary Table 1). Among the 192 miRNAs detected in both groups, eight miRNAs (bta-miR-126-5p, bta-miR-129, bta-miR-140, bta-miR-188, bta-miR-219, bta-miR-345-3p, bta-miR-4523, and bta-miR-760-3p) were up-regulated in OF-sEV/Pregnant and one miRNA (bta-miR-331-5p) was up-regulated in OF-sEV/Non-pregnant (*p* < 0.05; Figure 2).

**Figure 3:**
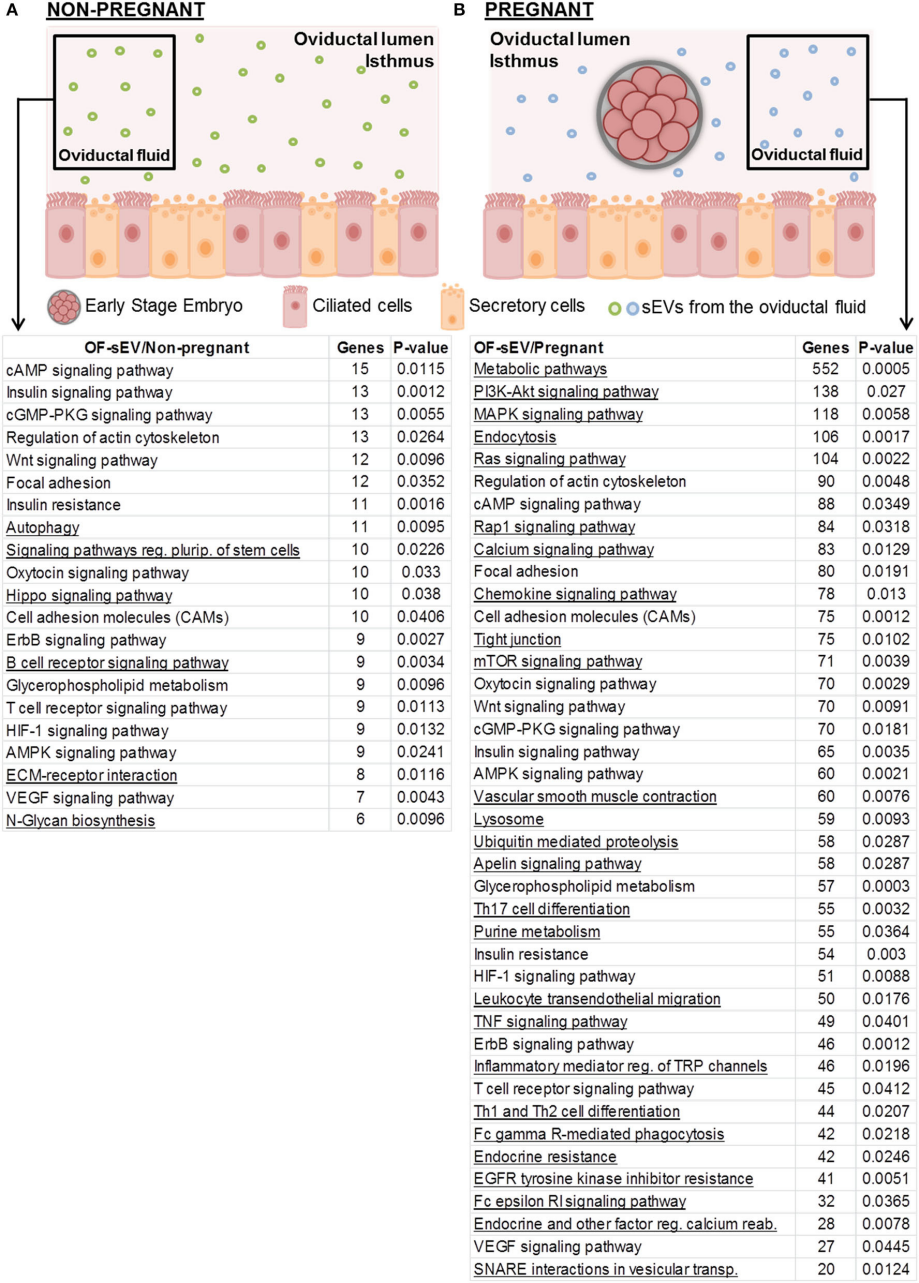
Selected biological pathways predicted to be regulated by miRNAs with increased expression in OF-sEVs. **(A)** Pathways predicted as regulated by the miRNA (bta-miR-331-5p) up-regulated in OF-sEV/Non-pregnant. **(B)** Pathways predicted as regulated by the eight miRNAs (bta-miR-126-5p, bta-miR-129, bta-miR-140, bta-miR-188, bta-miR-219, bta-miR-345-3p, bta-miR-4523, and bta-miR-760-3p) up-regulated in OF-sEV/Pregnant. Pathways were selected based on the potential relevance for oviductal functions and *p* < 0.05. Underlined pathways are modulated only in the group that they are present.

To investigate the biological functions of miRNAs differently expressed in OF-sEVs, we performed bioinformatics analysis. All the analyses were performed with up-regulated miRNAs. Based on their potential relevance for the oviductal milieu, 21 pathways were predicted to be modulated by the miRNA up-regulated in OF-sEVs from the non-pregnant group (Figure 3A), and 41 pathways predicted to be modulated by the miRNAs up-regulated in OF-sEVs from the pregnant group (Figure 3B) were selected. Our results demonstrated that differentially expressed OF-sEVs miRNAs modulate different pathways, such as PI3K-Akt signaling pathway, MAPK signaling pathway, and mTOR signaling pathway. For all identified pathways (*p* < 0.05), see Supplementary Tables 2, 3.

### miRNA Contents of OECs and Bioinformatics Analyses

**Figure 4:**
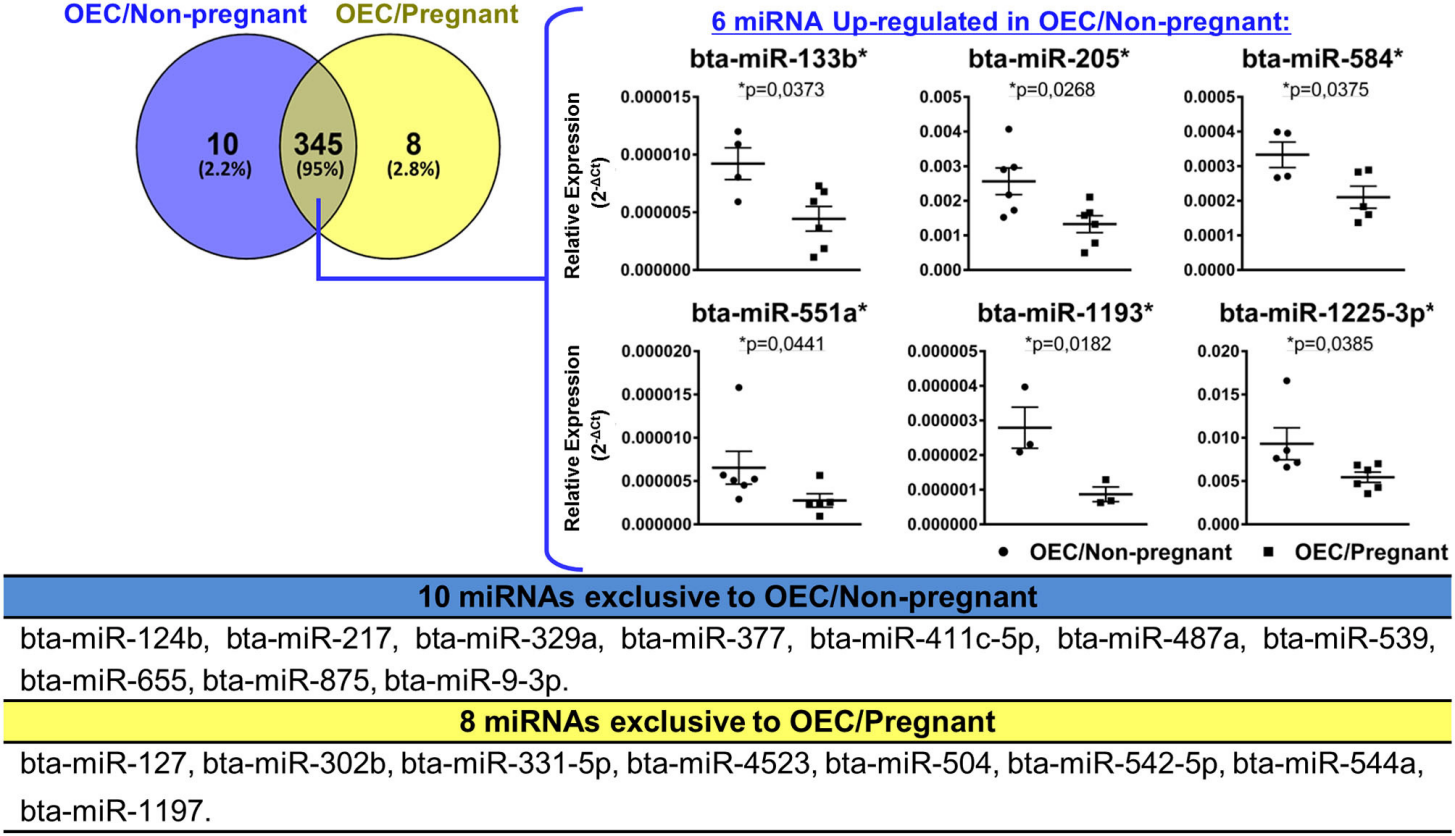
miRNAs profile of OECs from non-pregnant and pregnant cows. Venn diagram representing the detection of 363 miRNAs: 345 common between the two groups, 10 exclusive to OEC/Non-pregnant and eight exclusive to OEC/Pregnant. Among the 345 miRNAs detected in both groups, six miRNAs (bta-miR-133b, bta-miR-205, bta-miR-584, bta-miR-551a, bta-miR-1193, and bta-miR-1225-3p) were up-regulated in OEC/Non-pregnant. The asterisk (*) indicates miRNAs with relative expression significantly different (*p* < 0.05) within the groups. Samples size were *n* = 6 for both groups. Error bars represent SEM.

Next, we evaluated the levels of 383 miRNAs in OECs from the non-pregnant group (OEC/Non-pregnant) and the pregnant group (OEC/Pregnant). We identified a total of 355 and 353 miRNAs in OEC/Non-pregnant and OEC/Pregnant, respectively (for CT levels, see Supplementary Table 4). Of these, 345 miRNAs were commonly detected between the two groups, 10 were exclusive to OEC/Non-pregnant, and eight were exclusive to OEC/Pregnant. Among the 345 miRNAs detected in both groups, six miRNAs (bta-miR-133b, bta-miR-205, bta-miR-584, bta-miR-551a, bta-miR-1193, and bta-miR-1225-3p) were up-regulated in OEC/Non-pregnant, and none was up-regulated in OEC/Pregnant group and (*p* < 0.05; Figure 4).

**Figure 5:**
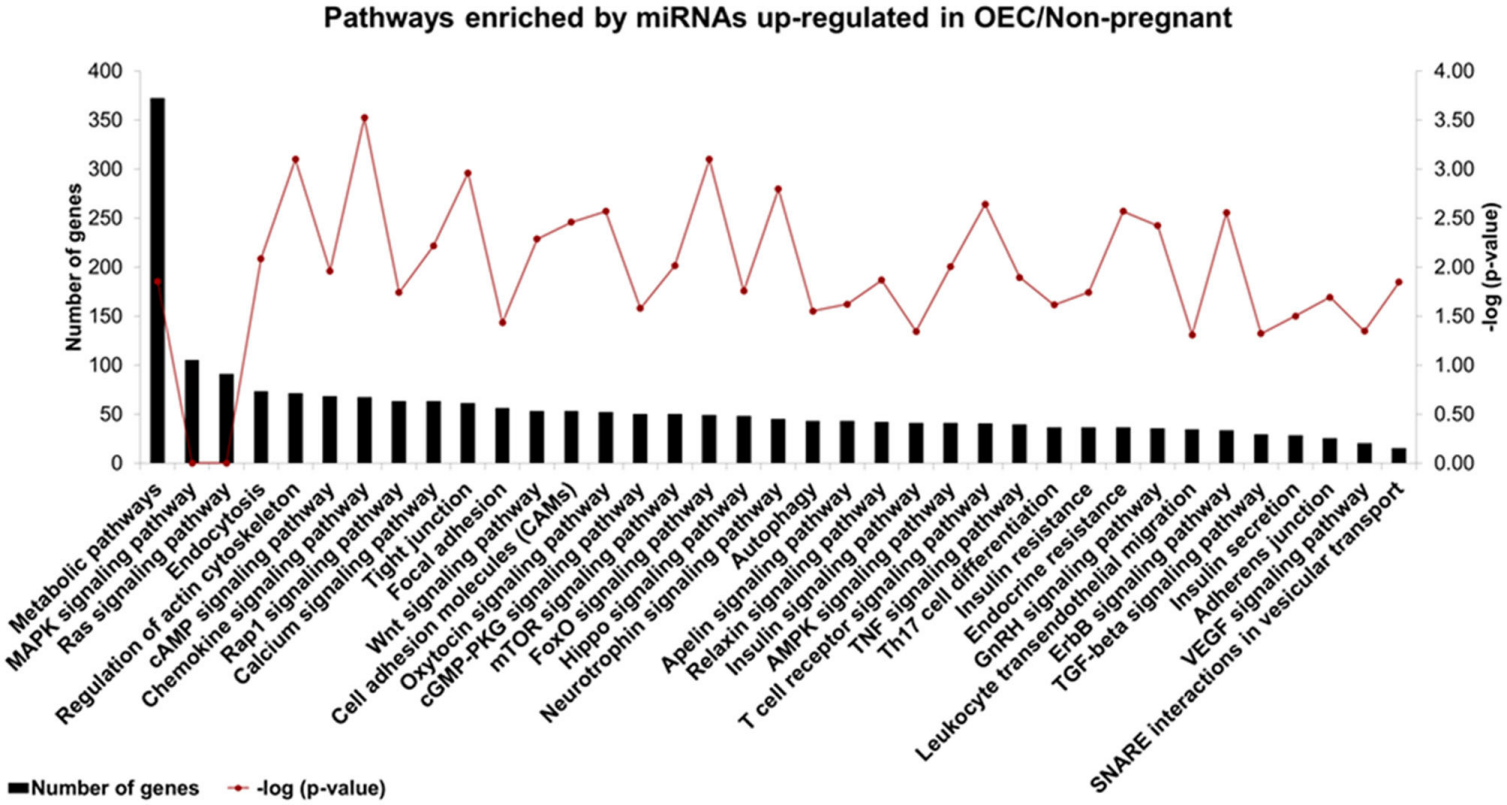
Biological pathways predicted using up-regulated miRNAs in OECs. Pathways predicted as regulated by six miRNAs (bta-miR-133b, bta-miR-205, bta-miR-584, bta-miR-551a, bta-miR-1193, and bta-miR-1225-3p) up-regulated in OECs from non-pregnant compared with OECs from pregnant cows. The left Y-axis values represent the number of genes predicted as modulated by the miRNAs for the respective pathways. The right Y-axis represents the enrichment score (–log10 of the *p*-value) for each pathway.

The prediction of biological functions modulated by miRNAs differently detected in OECs demonstrated 37 selected pathways predicted as regulated by six miRNAs (bta-miR-133b, bta-miR-205, bta-miR-584, bta-miR-551a, bta-miR-1193, and bta-miR-1225-3p) up-regulated in OECs from non-pregnant compared with pregnant cows (Figure 5). Our results suggest that differentially expressed OECs miRNAs modulate pathways, such as chemokine, oxytocin, and TNF signaling pathways. For all identified pathways (*p* < 0.05), see Supplementary Table 5.

### Relative Transcripts Levels for Selected Genes

**Figure 6:**
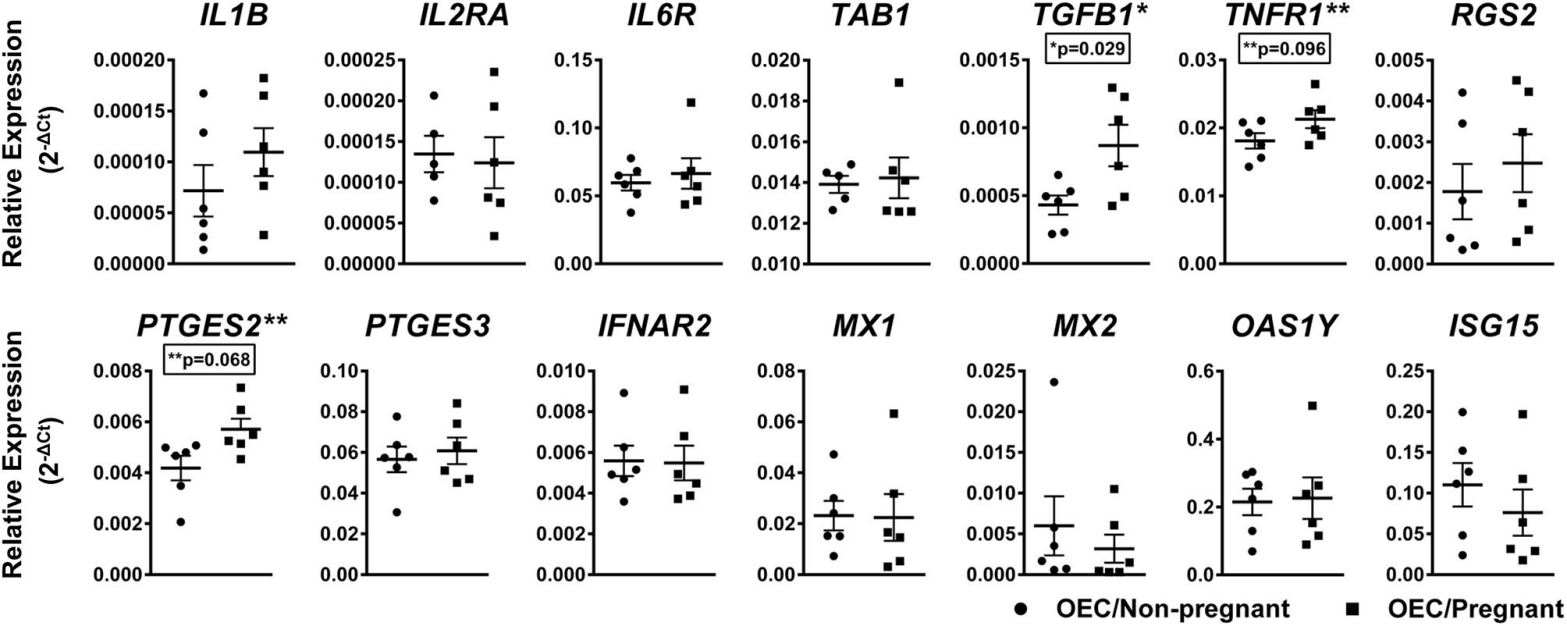
Relative mRNA expression of selected genes in OECs from non-pregnant compared with pregnant cows. Relative expression of up and downregulated mRNAs are shown as mean of 2^−ΔCt^ ± SEM. One asterisk (*) indicates genes with *p* < 0.05 and two asterisks (**) indicates genes with *p* < 0.1 within the groups. Samples size were *n* = 6 for both groups.

To evaluate genes present in predicted pathways regulated by miRNAs differently expressed in OECs, we analyzed the relative levels of 14 mRNAs selected from inflammatory, immune response, and pregnancy recognition pathways, known to influence oviductal function during early pregnancy. Considering a significant difference of *p* < 0.1, the genes *TGFB1* (*p* = 0.029), *TNFR1* (*p* = 0.096), and *PTGES2* (*p* = 0.068) were up-regulated in OECs from the pregnant group (Figure 6). Thus, our results demonstrate that the presence of one single embryo can affect the gene expression of those pathways in OECs.

## Discussion

Here we identified the miRNA contents of OF-sEVs and OECs as well as selected genes in OECs from pregnant and non-pregnant cows. This study indicates that communication within the pregnant animals, mediated by sEVs, is potentially initiated in the oviduct and, although the passage of spermatozoa and presence of a single embryo does not interfere with the OF-sEVs concentration, it modulates the miRNAs' contents in both OF-sEVs and OECs, as well as the transcript levels of the selected genes *TGFB1, TNFR1*, and *PTGES2* in OECs.

Oviductal flushing samples from the isthmus of ipsilateral oviducts were collected and used for sEVs isolation. The validation of the OF-sEVs isolation was based on the Minimal information for studies of extracellular vesicles 2018 [MISEV 2018; (28)]. No differences were identified in mode size or mean particle concentration between the groups; additionally, our results showed a population of vesicles with the expected size for sEVs, <200 nm (23). For instance, the passage of the spermatozoa and embryo presence do not induce changes in the oviductal secretion of sEVs, and the amount of sEVs secreted by the embryo were not enough to cause a detectable increase in the total amount of sEVs. Although there is no influence on the concentration of sEVs in pregnant cows, we were able to identify miRNAs with distinct levels between the pregnant and non-pregnant groups in both OF-sEVs and OECs.

In OF-sEVs, we identified eight miRNAs up-regulated in pregnant and one miRNA up-regulated in non-pregnant cows. The presence of EVs in the bovine oviductal fluid and their functional effects on the embryo are already recognized (30, 31, 66). miRNAs from oviductal EVs have been studied in bovines and murine across the estrous cycle, and in both models, it was showed a hormonal effect on the miRNAs in cyclic animals (32, 67). We showed that the passage of the spermatozoa and embryo presence could induce differential secretion of miRNAs in OF-sEVs. Among the up-regulated miRNAs in the pregnant group, the bta-miR-140 was also detected across the bovine estrous cycle, without differences among the stages (32). In the same study, the miRNAs bta-miR-129, bta-miR-188, bta-miR-219, bta-miR-345-3p, bta-miR-4523, bta-miR-760-3p identified in our study as up-regulated in pregnant cows were not detected across the estrous cycle. Similarly, the bta-miR-331-5p, up-regulated in our study in the non-pregnant group, was not detected across the estrus cycle (32). Thus, these differences in miRNA contents could be due to differences in the isolation protocol and mainly in the technique used for miRNAs analysis and not necessarily that these miRNAs originated from the embryo itself since they are also present in low levels in the non-pregnant cows.

Differentially expressed miRNAs in OF-sEVs modulate pathways, such as PI3K/AKT, mTOR, and MAPK, which are predicted to be modulated by up-regulated miRNAs from OF-sEVs from the pregnant group. These pathways are related to transcription, translation, proliferation, growth, control of the cytoskeletal organization, metabolism, and survival (68–72). Previous studies have demonstrated that PI3K/mTOR signaling pathways are related to angiogenesis, a crucial process in the female's reproductive system and essential for pregnancy establishment (73, 74). Additionally, PI3K/AKT pathway can mediate cell growth and survival in pre-implantation embryos. In early mouse embryos, PI3K-Akt subunits are expressed from zygote to blastocyst stage and are involved with blastomere proliferation (75, 76). In bovine pre-implantation embryos, the activation PI3K signaling is also required for apoptosis control (71). Together, these studies demonstrate that the presence and function of the PI3K/AKT/mTOR pathway are essential for embryo development. We suggest that these miRNAs within sEVs could influence embryo development in the oviduct, mediating maternal-embryonic communication during early embryo development. In order to test that, in future studies we aim to utilize oviductal sEVs collected from oviductal fluid from the pregnant group as a tool to modulate the embryo environment *in vitro*, as well as to study the effects of those sEVs on embryo quality and development.

In OECs, six miRNAs were up-regulated in the non-pregnant group (bta-miR-133b, bta-miR-205, bta-miR-584, bta-miR-551a, bta-miR-1193, and bta-miR-1225-3p) and none in the pregnant group. It is well-known that sex-steroid hormones, E2 and P4, coordinate changes in the oviductal transcriptome and secretory function across the estrous cycle (6, 77–79). Also, considering day 4 of the estrous cycle, the POF and CL size potentially affect the oviductal environment (50). In our results, no differences were found in POF and CL size as well as no differences in E2 and P4 between our groups, ensuring that they did not interfere with our results. The miRNAs bta-miR-133b, bta-miR-205, bta-miR-584, bta-miR-551a, identified in our study as up-regulated in non-pregnant cows, were already detected on bovine OECs exposed to low and high E2/P4 levels without the presence of the embryo, but with no differences in transcript levels (56). Thus, these miRNAs are not modulated by steroid hormones, supporting our data indicating that the passage of the spermatozoa and embryo presence can induce changes in miRNAs within oviductal cells in the same stage of the estrous cycle. Taken together, our results suggest that the passage of the spermatozoa and presence of an embryo within the oviduct can modulate the oviductal microenvironment, specifically the contents of miRNAs in OF-sEVs and OECs.

Based on bioinformatics analysis, we decided to evaluate the modulation of key-genes transcripts involved in the immune system, inflammation, and pregnancy recognition on OECs. Our study suggests that pregnancy stimulates changes in *TGFB1, PTGES2*, and *TNFR1* levels. TGFB1 is an anti-inflammatory cytokine present in OECs throughout the estrous cycle and crucial for tolerance to non-self-cells (80). Increased expression level of *TGFB1*, as found in our pregnant group, is also present in bovine OECs cultured with embryos *in vitro* (81). However, it cannot be disregarded that the increase in TGFB1 levels is also related to sperm binding and induction of sperm-tolerance (80, 82). Synergistically with *TGFB1*, the *PTGES2*, up-regulated in our pregnant group, participates in the eicosanoid metabolic process that will drive the production of PGE2 (83), promoting immune tolerance in the bovine oviduct (81, 84). Talukder et al. (81) and Maillo et al. (13) suggested that the embryo generates an anti-inflammatory response by suppressing key factors, such as NFkB2 and NFkBIA. The activation of NFkB transcriptional responses can be triggered by TNFR1 (85). In our results, *TNFR1* was increased in OECs from the pregnant cows. The TNFR1 is the receptor for the pro-inflammatory cytokines TNF-α. However, this cytokine also stimulates the productions of contractive substances, such as prostaglandins, regulating gametes transport, and ensuring the embryo migration into the uterus (86). Thus, it is imperative the adequate modulation of *TNFR1*, and among the modulators of the inflammatory responses of mammals are miRNAs (87). We identified a classic relationship between miR-584 and its predicted target TNFR1 in OECs. The bta-miR-584 was down-regulated in OECs from the pregnant group, while TNFR1 is increased in this group, while the opposite was observed in the non-pregnant group. Our results suggest that miRNAs may be decisive for the modulation of pro- and anti-inflammatory cytokines, leading to a proper immune response. Regarding genes involved in pregnancy recognition, no differences were identified between OECs groups. These results can be explained as the OECs from the pregnant group were exposed to embryos between 4 and 16 cells stage, and not morulas our blastocysts. Thereby, the interferon tau mechanism might not be initiated during this stage. Similar results were also found *in vitro*, where the presence of day-04 embryo did not influence the expression of interferon-stimulated genes (such as *ISG15, OAS1, MX2*) and *IFNAR2* on bovine OECs (81). Taken together, pregnancy can dynamically modulate the immune system and inflammation response as early as day 4, affecting the oviductal environment and possibly embryo survival and development.

In this study, we identify the modulation of miRNAs and mRNAs in the oviduct. However, some limitations should be noticed since our experimental model cannot determine if differently expressed miRNAs are a direct or indirect effect of the spermatozoa or the embryo. Nevertheless, previous studies have shown that the embryo has a local effect on both, the oviduct and the endometrium (13, 62, 88). Additionally, the miRNAs up-regulated in OF-sEVs from the pregnant group are potentially secreted by the embryo, by the oviduct as a result of the embryonic stimulus, or from both. Although the ideal model to isolate the embryo effect is to transfer embryos directly into the oviduct (13), we must not disregard that the physiological environment is dynamic and depends on a set of synchronous factors and signals that together will guarantee the success of the pregnancy (89). Therefore, we chose not to remove these factors or to amplify the embryo's signal by transferring a large number of embryos as it would not represent the physiological response of the oviductal milieu. Additionally, future approaches should consider increase the number of animals and also small RNA sequencing analysis for a more comprehensive understanding of the expression and regulation of miRNAs associated with pregnancy in OECs and sEVs.

In conclusion, our results demonstrate that pregnancy can modulate the miRNA contents of sEVs as well as the miRNAs and mRNAs levels in OECs in cows. The functional enrichment of miRNAs highlights pathways involved in physiological processes, such as inflammation, cell proliferation, and immune response, crucial for both the reproductive tract and embryo development. Furthermore, our results confirm *in vivo* that the embryo-maternal crosstalk starts within the oviduct, enhancing oviductal receptivity, allowing proper fertilization and early embryo development, thus leading to a successful pregnancy.

## Data Availability Statement

The original contributions presented in the study are included in the article/Supplementary Material, further inquiries can be directed to the corresponding author/s.

## Ethics Statement

The animal study was reviewed and approved by University of São Paulo Research Ethics Committee (Protocol Number 4909010817).

## Author Contributions

RM and JS designed the study. RM, NB, AB, MC, GA, and CP collected the samples and performed the experiments. RM, FP, and JS analyzed the data and wrote the manuscript. JP and GP performed the ultrasonography and artificial insemination. LS provided the conditions for cattle housing and management. FM and GP contributed the expertise and reagents. All authors discussed, reviewed, and agreed to the final version of the manuscript.

## Conflict of Interest

The authors declare that the research was conducted in the absence of any commercial or financial relationships that could be construed as a potential conflict of interest.
