# Supplementary material for: Changes in Oviductal Cells and Small Extracellular Vesicles miRNAs in Pregnant Cows
Source: Front Vet Sci. 2021 Mar 4;8:639752. doi: 10.3389/fvets.2021.639752 (PMC7969882; doi:10.3389/fvets.2021.639752)
Supplement: Supplementary file 1 [file Data_Sheet_1.PDF]

Supplementary Data 1

The isolation of OF-sEVs was confirmed using transmission electron microscopy (TEM) and western blotting analysis. For the western blotting analyses, see DE ÁVILA et al. (2020). Due to the small volume of oviductal flush obtained from a single animal, the TEM was performed with oviducts obtained from slaughterhouses. In order to be as close as possible to our animal model, only reproductive tracts with a CL classified as stage 1 (early corpus luteum, red and recently ovulated), according to IRELAND et al. (1980), were used. The isthmus of the ipsilateral oviducts was flushed, and the OF-sEVs were obtained as previously described.

SEVs were isolated from 3 mL of oviductal flush and according to the methods described by DE ÁVILA et al. (2020), with minimal modifications. The obtained pellet was diluted in 50µL of fixation solution (1% sodium cacodylate; 2% glutaraldehyde; 2% paraformaldehyde; pH 7.2) for 2h at room temperature. After that, the OF-sEVs were diluted in 2 mL of PBS and centrifuged (119700 xg, 70 min, 4 °C) in order to remaining fixative. The pellet obtained was diluted in 20 µL of buffer solution (1% sodium cacodylate; pH 7.2) and kept refrigerated until analysis. The contents were placed in a copper grid for 20 min at room temperature to air dry. Subsequently, a drop of 2% uranyl acetate, which enhances the contrast, was added to the grid and examined in a FEI Tecnai 20 (LAB6 emission) at 200 KV. Through the images, we were able to identify cup-shaped particles with characteristic size resemble sEVs (Supplementary Figure 1). Additionally, through the western blotting analyses performed by DE ÁVILA et al. (2020), our isolation protocol generates an enriched pallet with sEVs and with no cell contamination.

.
