# Supplementary material for: Changes in Oviductal Cells and Small Extracellular Vesicles miRNAs in Pregnant Cows
Source: Front Vet Sci. 2021 Mar 4;8:639752. doi: 10.3389/fvets.2021.639752 (PMC7969882; doi:10.3389/fvets.2021.639752)
Supplement: Supplementary file 2 [file Table_1.pdf]

S1 Table. Cycle threshold levels of the 261 miRNAs detected in OF-sEVs isolated from oviductal flushes from non-pregnant (OF-sEV/Non-pregnant) and pregnant (OF-sEV/Pregnant) and cows.

	**MicroRNAs**	**Group**											
		**OF-sEV/Non-pregnant**						**OF-sEV/Pregnant**					
		**1**	**2**	**3**	**4**	**5**	**6**	**1**	**2**	**3**	**4**	**5**	**6**
**(n)**	**Detected in sEV/Non-pregnant**												
1	**bta-let-7a-3p**	35.94	.	.	.	34.89	31.82	.	34.30	.	.	.	.
2	**bta-miR-107**	34.43	36.91	.	.	.	34.60	33.46	.	34.58	.	.	.
3	**bta-miR-10b**	33.84	34.90	33.93	35.80	.	33.47	32.08	.	34.50	.	.	.
4	**bta-let-7i**	33.54	36.11	33.42	.	34.53	34.51	30.16	34.52	.	.	.	.
5	**bta-miR-124a**	34.29	35.33	.	.	32.97	36.64	32.37	34.31	.	.	.	.
6	**bta-miR-101**	31.55	.	33.99	34.88	.	31.74	30.73	34.82	.	.	.	.
7	**bta-miR-128**	34.76	.	36.98	.	.	34.17	32.41	.	.	.	.	.
8	**bta-miR-139**	33.43	.	.	34.22	34.64	.	31.59	.	34.46	.	.	.
9	**bta-miR-133a**	32.81	35.15	34.64	.	34.73	.	30.76	.	.	.	.	34.95
10	**bta-miR-145**	29.72	31.24	31.12	30.98	31.93	33.89	.	.	30.05	.	.	.
11	**bta-miR-15a**	30.73	.	34.64	.	36.12	.	30.06	.	33.95	.	.	.
12	**bta-miR-149-5p**	33.46	32.46	35.67	33.48	35.02	34.91	29.83	35.03	.	.	.	.
13	**bta-miR-181a**	34.64	.	34.02	.	34.50	34.34	30.46	.	34.00	.	.	.
14	**bta-miR-192**	34.43	.	34.38	36.35	33.29	.	31.92	.	34.27	.	.	.
15	**bta-miR-185**	32.56	34.58	33.93	33.84	33.94	35.33	29.78	.	35.96	.	.	.
16	**bta-miR-193b**	32.94	34.90	35.29	35.84	33.68	.	32.83	34.92	.	.	.	.
17	**bta-miR-196b**	35.41	.	33.52	36.79	32.91	35.89	35.18	.	.	.	33.20	.
18	**bta-miR-19b**	33.52	.	32.90	.	33.43	31.77	33.28	.	34.13	.	.	.
19	**bta-miR-21-5p**	34.89	.	34.12	34.98	.	.	.	.	.	36.69	.	.
20	**bta-miR-212**	.	.	35.26	35.18	.	33.96	.	.	.	.	33.68	.
21	**bta-miR-216a**	37.00	35.41	.	.	36.48	.	31.73	.	.	.	.	35.91
22	**bta-miR-218**	35.28	36.12	.	.	.	35.46	.	.	.	.	.	.
23	**bta-miR-27a-5p**	32.94	.	33.72	36.30	34.00	.	32.60	.	35.63	.	.	.
24	**bta-miR-301b**	35.55	35.94	35.94	.	35.89	.	31.83	.	35.93	.	.	.
25	**bta-miR-330**	33.53	34.15	36.77	33.64	.	35.32	30.49	.	.	.	.	.
26	**bta-miR-331-3p**	34.80	.	.	34.59	.	33.92	31.76	.	.	.	.	.
27	**bta-miR-338**	34.86	35.90	.	35.97	.	.	32.84	.	.	.	.	.
28	**bta-miR-324**	34.91	33.33	34.06	.	.	.	31.11	.	.	.	.	.
29	**bta-miR-365-3p**	32.88	.	33.34	34.39	34.32	34.09	32.76	.	33.10	.	.	.
30	**bta-miR-342**	33.51	34.89	.	34.23	.	.	32.88	.	34.31	.	.	.
31	**bta-miR-369-5p**	34.50	33.86	35.03	36.03	33.78	.	.	.	33.93	.	.	.
32	**bta-miR-374b**	33.24	.	34.14	35.97	.	33.03	31.87	.	.	.	.	.
33	**bta-miR-362-3p**	.	.	34.48	35.89	34.06	34.78	33.77	.	36.93	.	.	.
34	**bta-miR-376a**	36.79	33.63	35.19	.	.	.	34.04	.	.	.	.	.
35	**bta-miR-376d**	32.51	33.35	32.26	.	.	.	36.00	.	.	.	.	.
36	**bta-miR-376e**	32.13	33.51	32.83	.	.	.	.	.	33.95	.	.	.
37	**bta-miR-380-5p**	.	36.34	34.95	33.93	32.82	33.02	.	.	35.73	.	.	.
38	**bta-miR-449d**	32.44	33.11	32.81	32.84	32.56	33.97	.	.	32.50	.	.	34.97
39	**bta-miR-424-5p**	31.42	36.62	32.87	.	36.07	32.81	29.70	33.24	.	.	.	.
40	**bta-miR-452**	.	.	.	34.90	34.81	33.28	33.76	.	.	.	.	.
41	**bta-miR-431**	32.63	34.92	33.27	35.32	33.78	35.30	.	35.67	32.88	.	.	.
42	**bta-miR-432**	35.57	34.10	.	35.56	35.85	.	31.82	.	33.10	.	.	.
43	**bta-miR-497**	32.76	34.85	34.78	33.84	33.85	.	28.73	.	33.24	.	.	.
44	**bta-miR-488**	33.95	34.98	.	35.55	.	.	30.87	.	32.86	.	.	.
45	**bta-miR-490**	.	35.64	.	34.39	31.78	.	30.81	.	.	.	.	.
46	**bta-miR-491**	33.01	.	.	34.66	32.37	33.14	30.43	.	34.69	.	.	.
47	**bta-miR-532**	32.85	33.15	31.61	36.49	33.95	33.97	30.73	.	31.80	.	.	.
48	**bta-miR-652**	32.76	34.26	35.45	.	.	33.38	.	.	35.41	.	.	.

49	**bta-miR-658**	33.18	34.62	34.59	34.31	.	.	31.70	.	33.98	.	.	.
50	**bta-miR-660**	32.52	36.37	.	.	34.03	.	31.76	.	.	.	.	35.76
51	**bta-miR-665**	29.73	29.69	31.78	30.80	31.66	34.09	.	.	.	.	32.89	.
52	**bta-miR-671**	34.14	36.22	.	35.25	35.06	.	.	.	.	.	.	35.61
53	**bta-miR-769**	33.33	35.88	.	.	.	33.55	.	.	.	.	.	.
54	**bta-miR-708**	33.61	35.59	33.94	34.32	34.27	.	31.83	.	34.42	.	.	.
55	**bta-miR-744**	33.32	.	.	.	32.97	35.03	.	35.21	.	.	.	35.16
56	**bta-miR-1306**	36.84	33.31	.	33.10	33.40	34.33	30.77	.	.	36.85	.	.

(n)	Detected in OF-sEV/Pregnant												
1	**bta-miR-133b**	.	.	.	.	.	.	36.91	.	36.25	.	33.48	.
2	**bta-miR-147**	.	.	.	.	.	32.10	32.01	35.68	35.47	.	.	33.41
3	**bta-miR-196a**	.	.	.	.	33.24	33.98	.	.	.	36.67	31.41	33.96
4	**bta-miR-215**	34.68	.	.	36.01	.	.	32.94	.	35.05	.	35.87	34.80
5	**bta-miR-23b-5p**	.	.	.	.	34.33	32.63	33.65	.	.	.	32.00	33.34
6	**bta-miR-29d-5p**	34.92	.	.	.	.	34.04	32.00	35.80	35.90	.	.	36.61
7	**bta-miR-29e**	33.94	.	34.39	.	.	.	32.57	34.63	34.12	.	.	.
8	**bta-miR-371**	34.23	35.64	.	.	.	.	32.57	.	33.99	.	36.49	.
9	**bta-miR-409a**	33.94	.	.	34.92	.	.	35.97	.	35.97	.	36.14	35.36
10	**bta-miR-409b**	.	.	.	.	.	.	36.65	.	35.86	.	36.80	.
11	**bta-miR-379**	.	.	.	.	.	.	34.00	34.45	37.00	.	.	.
12	**bta-miR-483**	.	.	34.91	.	.	.	33.88	36.64	35.49	.	.	.
13	**bta-miR-670**	34.35	.	.	.	.	34.14	35.41	.	34.54	.	32.98	.

(n)	Detected in OF-sEV/Pregnant and OF-sEV/Non-pregnant												
1	**bta-miR-103**	30.75	35.84	33.33	33.40	31.71	32.84	28.28	36.54	31.85	.	.	34.39
2	**bta-let-7a-5p**	27.51	31.69	30.18	.	29.59	28.72	26.55	30.67	30.34	35.03	33.48	33.95
3	**bta-let-7b**	26.74	28.91	28.76	28.75	27.75	28.35	23.65	29.17	27.60	33.81	33.83	31.69
4	**bta-miR-105b**	35.11	34.12	35.00	33.99	33.62	.	30.99	33.95	34.10	.	.	.
5	**bta-let-7c**	26.74	29.50	29.43	29.01	28.02	27.75	24.31	29.15	27.69	34.40	33.85	31.78
6	**bta-miR-106a**	29.69	34.77	31.82	33.65	30.64	30.84	29.09	33.26	33.50	.	33.15	32.66
7	**bta-let-7d**	28.73	33.96	32.06	31.89	29.96	30.29	27.70	30.79	31.11	.	.	.
8	**bta-miR-106b**	30.60	32.18	33.41	32.99	32.81	32.85	28.16	32.92	.	.	31.97	32.64
9	**bta-let-7e**	26.96	30.39	29.83	29.75	28.09	27.83	25.81	29.61	29.23	34.15	.	33.08
10	**bta-let-7f**	29.56	35.05	31.89	33.48	31.71	30.70	28.80	31.74	32.16	.	34.76	35.47
11	**bta-miR-10a**	31.71	33.97	34.38	.	34.68	34.15	30.81	36.78	35.63	.	.	32.88
12	**bta-let-7g**	30.77	.	34.26	.	33.36	32.40	30.82	34.07	32.79	.	34.93	.
13	**bta-miR-122**	.	.	33.45	35.97	33.03	35.94	36.15	35.11	35.20	34.47	.	.
14	**bta-miR-100**	30.51	32.59	31.86	33.99	32.87	33.06	29.05	.	34.16	.	34.94	35.60
15	**bta-miR-124b**	33.92	.	.	35.56	32.87	.	32.18	.	.	.	33.93	33.64
16	**bta-miR-125a**	30.12	34.81	32.03	32.88	31.92	31.79	27.69	34.78	33.36	36.51	35.68	.
17	**bta-miR-125b**	27.75	32.09	29.64	30.22	29.27	28.78	25.73	30.65	29.97	34.45	34.81	33.48
18	**bta-miR-126-3p**	31.64	31.88	31.38	31.73	32.16	31.68	31.56	32.62	31.63	32.35	31.90	32.86
19	**bta-miR-126-5p**	33.05	35.67	35.42	35.72	33.97	34.04	31.43	32.91	35.68	36.03	33.41	32.86
20	**bta-miR-134**	36.13	.	.	35.18	35.44	.	31.34	35.17	33.14	.	.	.
21	**bta-miR-127**	27.92	28.82	29.81	28.86	29.64	31.90	25.10	29.40	28.21	33.44	31.81	31.32
22	**bta-miR-135a**	30.30	34.74	32.80	32.71	32.35	32.51	28.75	34.26	32.75	.	.	.
23	**bta-miR-135b**	30.71	.	33.98	32.90	33.94	32.74	29.79	34.62	33.27	.	.	.
24	**bta-miR-129**	33.60	.	35.96	34.70	.	33.95	29.72	33.05	33.94	.	.	35.54
25	**bta-miR-129-3p**	34.45	.	.	36.72	33.89	.	32.86	.	34.97	.	33.97	35.26
26	**bta-miR-129-5p**	32.02	34.88	34.62	.	32.31	.	29.42	.	32.29	.	.	36.94
27	**bta-miR-138**	34.86	34.45	35.19	33.93	33.56	34.12	29.68	.	.	.	34.25	33.63
28	**bta-miR-130a**	31.33	31.52	31.79	31.55	31.64	32.98	30.74	31.40	31.73	32.19	32.23	32.61
29	**bta-miR-130b**	30.12	30.75	31.44	.	30.49	31.59	28.34	30.74	30.03	32.65	31.56	32.19
30	**bta-miR-140**	33.31	.	33.85	35.06	33.04	.	31.35	32.89	32.40	.	32.62	.
31	**bta-miR-132**	33.41	.	34.03	34.72	34.29	32.94	33.78	35.04	35.01	.	34.99	35.52

32	**bta-miR-141**	28.90	31.85	30.48	.	30.74	30.84	27.49	32.61	.	.	33.82	32.37
33	**bta-miR-151-3p**	31.49	33.94	35.00	33.93	32.84	.	29.66	34.36	32.75	.	26.48	33.87
34	**bta-miR-143**	32.68	34.26	32.91	32.66	30.70	30.62	31.60	32.90	31.84	35.14	32.31	31.68
35	**bta-miR-151-5p**	31.11	.	33.67	34.36	32.35	.	29.67	36.17	32.28	.	33.69	34.94
36	**bta-miR-152**	32.93	.	35.45	.	36.14	.	32.59	35.99	35.01	.	.	.
37	**bta-miR-154a**	.	34.07	33.08	35.96	35.71	.	31.73	35.52	35.95	36.89	.	.
38	**bta-miR-154b**	31.39	30.82	30.35	29.84	29.82	.	30.70	31.88	30.56	30.05	29.46	30.66
39	**bta-miR-154c**	34.38	.	36.01	34.80	36.42	.	34.06	34.08	35.01	36.80	35.48	34.53
40	**bta-miR-148a**	27.96	31.07	30.50	30.33	29.31	28.79	27.20	32.82	30.91	33.91	.	34.92
41	**bta-miR-148b**	28.45	33.97	30.74	32.15	30.30	29.44	27.74	31.90	31.79	35.96	35.11	35.21
42	**bta-miR-149-3p**	25.77	26.24	27.73	26.78	28.58	31.09	.	27.47	25.55	33.32	32.27	29.82
43	**bta-miR-15b**	31.87	34.74	34.29	33.31	32.89	.	30.45	33.87	32.91	36.37	34.73	.
44	**bta-miR-16a**	30.43	32.98	32.68	33.00	31.59	.	29.50	33.96	33.32	.	33.87	33.25
45	**bta-miR-16b**	29.64	33.66	31.40	33.73	31.37	.	28.76	32.86	32.61	.	33.98	32.94
46	**bta-miR-188**	33.80	34.05	36.90	36.50	34.79	.	30.71	36.28	33.96	35.82	32.26	32.39
47	**bta-miR-17-5p**	33.86	33.18	33.93	33.21	31.97	36.07	31.79	35.34	33.85	34.95	30.77	31.71
48	**bta-miR-181b**	31.96	34.43	.	.	33.08	34.82	30.56	.	35.26	.	32.93	.
49	**bta-miR-190b**	34.34	.	36.37	.	34.69	34.47	33.24	.	34.99	.	35.59	.
50	**bta-miR-181d**	32.50	33.54	35.10	.	32.56	35.23	30.09	.	32.51	.	.	35.71
51	**bta-miR-191**	30.75	33.70	33.85	34.04	32.13	31.64	.	32.29	32.99	17.17	33.15	32.91
52	**bta-miR-184**	35.04	34.51	35.42	.	.	..	33.41	34.14	33.88	.	.	.
53	**bta-miR-193a-5p**	31.31	33.84	32.35	34.17	33.41	33.06	29.50	33.91	32.72	.	.	36.03
54	**bta-miR-186**	33.11	34.61	35.07	33.62	36.68	34.39	31.49	.	34.54	.	.	35.32
55	**bta-miR-187**	30.60	32.70	31.24	31.88	30.52	31.85	28.76	32.94	32.08	.	37.00	32.86
56	**bta-miR-195**	30.46	33.93	31.18	32.78	31.81	33.52	29.26	33.44	33.16	32.85	33.00	.
57	**bta-miR-200c**	27.83	35.09	29.83	30.99	29.64	28.68	26.63	31.69	30.71	.	31.82	32.87
58	**bta-miR-204**	31.45	33.92	33.34	34.92	33.20	33.91	28.78	33.97	32.50	.	36.65	.
59	**bta-miR-197**	31.55	32.97	32.27	34.02	36.77	32.27	28.73	34.43	31.83	.	.	36.68
60	**bta-miR-205**	30.93	33.60	34.22	32.90	32.64	.	29.57	36.69	33.69	.	.	.
61	**bta-miR-206**	34.93	.	35.90	34.43	.	.	32.93	36.96	35.33	.	.	.
62	**bta-miR-199c**	33.25	34.68	33.29	32.13	36.93	33.55	29.67	32.91	34.01	.	35.12	35.10
63	**bta-miR-20a**	30.63	33.42	32.84	32.98	31.84	30.98	28.98	33.85	33.85	.	33.11	34.61
64	**bta-miR-19a**	31.84	.	.	34.70	32.13	31.81	33.71	33.86	34.03	.	.	.
65	**bta-miR-20b**	30.81	.	33.14	34.79	32.40	32.55	29.80	33.12	33.87	.	32.44	34.61
66	**bta-miR-200a**	31.73	.	31.67	33.68	33.84	32.68	29.80	32.29	33.93	.	.	35.72
67	**bta-miR-200b**	26.54	31.10	29.05	29.81	28.74	26.88	25.50	29.74	29.63	34.90	33.93	31.67
68	**bta-miR-210**	30.37	33.25	32.54	32.90	31.71	31.72	28.31	32.87	31.38	.	.	33.94
69	**bta-miR-211**	31.95	34.97	33.92	34.11	35.05	.	29.37	35.66	33.54	.	.	34.99
70	**bta-miR-22-5p**	32.77	34.27	33.09	.	36.03	36.10	32.14	33.68	.	.	.	34.04
71	**bta-miR-221**	31.90	30.74	32.67	32.89	32.11	.	28.48	34.50	30.53	.	36.50	34.93
72	**bta-miR-214**	29.47	31.91	31.59	.	31.54	34.16	26.05	31.54	29.32	.	.	33.94
73	**bta-miR-222**	30.70	30.57	34.55	31.58	31.36	33.06	26.63	33.33	30.28	.	.	35.18
74	**bta-miR-223**	33.42	34.15	.	.	33.99	.	32.00	35.64	32.90	.	.	.
75	**bta-miR-23a**	28.38	33.11	29.84	30.85	29.57	29.29	26.42	31.97	30.60	.	33.92	31.73
76	**bta-miR-23b-3p**	31.80	32.85	31.75	32.38	30.53	31.78	28.64	32.76	32.90	.	.	33.67
77	**bta-miR-219**	33.15	33.68	33.95	34.60	32.26	34.19	29.85	.	33.14	.	.	33.87
78	**bta-miR-219-3p**	33.96	36.09	35.77	36.33	.	36.82	32.01	36.49	35.05	.	.	32.87
79	**bta-miR-24-3p**	28.38	30.87	30.75	30.57	29.39	29.45	26.62	30.76	30.70	34.93	.	.
80	**bta-miR-25**	29.82	32.22	32.39	32.48	31.83	31.86	27.69	34.60	31.71	.	34.81	.
81	**bta-miR-22-3p**	3.45	2.72	2.93	.	2.66	2.68	3.40	2.92	3.08	2.93	2.54	2.69
82	**bta-miR-26a**	27.78	32.90	29.46	30.73	28.84	28.68	26.38	31.43	30.60	.	34.38	32.78
83	**bta-miR-26b**	31.60	.	31.26	33.90	34.88	30.72	29.26	34.27	34.29	.	34.96	34.91
84	**bta-miR-29d-3p**	30.74	35.06	31.89	34.00	32.64	32.86	28.61	35.59	31.57	.	.	33.88
85	**bta-miR-27a-3p**	30.03	35.29	32.23	31.53	31.58	30.61	29.17	33.00	32.53	.	35.05	35.21
86	**bta-miR-301a**	34.64	34.57	.	.	35.05	.	32.39	.	35.82	34.91	.	.
87	**bta-miR-27b**	30.78	.	31.61	32.57	32.85	30.81	29.23	.	33.34	.	.	33.50

88	**bta-miR-296-3p**	31.81	32.45	32.44	33.02	32.10	31.90	29.20	36.04	32.94	35.02	32.75	32.63
89	**bta-miR-296-5p**	32.21	27.80	32.69	32.34	32.94	.	30.71	29.01	27.63	.	35.38	32.85
90	**bta-miR-29a**	28.25	31.63	30.59	30.65	29.49	29.74	26.19	31.69	29.78	34.93	.	34.05
91	**bta-miR-29b**	30.04	33.90	32.42	34.16	33.27	32.59	29.40	34.45	33.89	.	.	.
92	**bta-miR-30a-5p**	31.54	34.57	32.87	34.54	32.21	32.24	29.77	35.23	35.18	.	34.82	32.88
93	**bta-miR-29c**	28.03	31.90	29.76	.	29.49	29.72	26.22	31.17	29.96	.	35.57	33.98
94	**bta-miR-30b-5p**	32.74	32.95	.	35.02	31.68	33.65	29.97	.	32.91	.	.	36.81
95	**bta-miR-328**	30.74	34.36	32.18	31.68	29.73	28.45	29.08	33.02	32.32	33.95	35.67	35.49
96	**bta-miR-30c**	30.51	.	31.87	33.06	31.83	31.90	28.70	33.05	32.48	.	34.90	34.17
97	**bta-miR-30d**	32.68	.	32.89	32.74	32.76	31.72	30.02	35.35	33.68	.	35.80	34.54
98	**bta-miR-30e-5p**	32.65	.	33.16	32.36	32.83	32.01	29.83	34.58	.	.	32.72	33.52
99	**bta-miR-30f**	31.68	.	35.85	34.96	32.87	32.89	29.77	33.19	33.45	.	.	.
100	**bta-miR-31**	29.41	32.89	31.60	31.54	29.52	30.49	27.33	32.65	31.38	.	32.21	.
101	**bta-miR-331-5p**	31.72	31.96	30.83	31.40	31.81	33.16	31.69	33.80	32.60	35.64	35.17	36.50
102	**bta-miR-335**	33.72	.	36.26	34.86	34.81	36.98	35.93	.	35.06	.	33.05	34.93
103	**bta-miR-320a**	25.80	27.33	27.60	26.80	27.67	30.18	23.15	27.90	26.59	33.88	31.98	29.81
104	**bta-miR-320b**	31.38	31.51	31.57	32.08	33.21	.	27.75	33.96	30.36	.	34.21	.
105	**bta-miR-339a**	31.81	33.96	36.25	32.86	.	35.00	28.82	35.03	32.23	.	.	.
106	**bta-miR-323**	17.41	17.64	17.83	16.71	16.76	17.46	17.28	17.41	17.36	18.73	17.05	16.76
107	**bta-miR-339b**	31.29	34.82	33.38	31.62	31.89	32.88	27.96	34.22	.	35.03	.	.
108	**bta-miR-326**	31.81	33.13	34.79	36.04	35.75	36.55	29.93	36.76	33.40	.	.	.
109	**bta-miR-33b**	34.00	33.89	33.79	34.89	36.48	.	34.33	33.66	34.32	35.90	35.51	.
110	**bta-miR-365-5p**	33.38	33.66	.	33.00	.	.	30.75	33.99	33.56	.	.	34.77
111	**bta-miR-345-3p**	33.98	36.08	35.88	34.15	.	.	.	33.88	34.00	.	.	35.40
112	**bta-miR-345-5p**	28.67	30.65	31.02	29.70	30.77	33.49	26.69	31.40	29.06	36.09	35.39	33.91
113	**bta-miR-346**	31.30	30.90	31.87	32.55	33.68	34.64	26.45	34.14	30.82	.	36.80	36.63
114	**bta-miR-34a**	33.42	.	33.93	33.98	.	34.13	31.80	36.89	33.47	.	.	.
115	**bta-miR-370**	32.86	33.87	34.15	33.86	33.98	.	30.77	36.58	34.99	34.81	36.54	33.43
116	**bta-miR-34b**	29.10	33.30	31.47	31.74	31.92	29.54	28.80	31.70	31.67	.	.	35.21
117	**bta-miR-34c**	29.63	34.23	31.78	31.62	30.84	30.27	28.60	32.24	31.72	.	.	.
118	**bta-miR-361**	32.31	34.45	33.83	34.34	34.84	33.57	31.03	36.38	34.87	.	.	.
119	**bta-miR-375**	30.18	31.41	31.50	31.06	31.51	32.93	28.48	31.66	31.14	31.87	31.60	33.71
120	**bta-miR-382**	29.05	28.83	29.43	29.83	30.07	29.84	29.66	29.79	28.73	31.54	30.51	31.41
121	**bta-miR-383**	27.56	30.09	30.75	36.00	36.48	.	32.11	33.24	31.71	.	.	.
122	**bta-miR-378**	32.86	33.19	33.66	34.82	33.10	31.65	29.80	.	33.84	.	34.05	.
123	**bta-miR-378b**	32.28	34.32	35.71	34.81	32.92	32.91	31.10	35.22	35.53	.	.	36.48
124	**bta-miR-411a**	32.52	31.88	32.74	32.79	32.62	31.66	.	32.90	32.49	34.15	32.80	31.66
125	**bta-miR-378c**	34.96	36.68	34.35	32.82	33.90	34.55	36.82	.	34.90	33.91	33.16	32.77
126	**bta-miR-411b**	36.43	.	34.90	33.85	33.84	34.36	31.24	.	35.71	.	32.96	35.25
127	**bta-miR-378d**	32.70	35.13	33.31	32.17	32.89	33.56	.	33.42	31.90	33.96	33.85	34.24
128	**bta-miR-421**	31.93	32.54	32.35	30.39	32.91	31.67	.	33.16	31.18	29.68	30.37	31.11
129	**bta-miR-423-3p**	30.17	31.77	34.74	31.88	31.57	31.78	28.06	32.33	31.53	.	.	.
130	**bta-miR-423-5p**	29.51	32.85	31.99	32.77	31.43	32.47	28.05	31.76	31.73	.	.	.
131	**bta-miR-449c**	33.81	34.91	33.74	34.05	.	35.62	30.53	36.31	34.42	.	.	.
132	**bta-miR-425-3p**	25.38	26.35	27.39	26.00	26.94	29.99	21.68	27.81	24.70	34.36	31.82	29.53
133	**bta-miR-425-5p**	31.33	36.68	33.98	34.36	33.02	32.28	29.26	34.05	33.30	34.92	.	.
134	**bta-miR-429**	29.48	30.19	29.70	29.48	30.55	30.83	28.81	29.50	29.61	32.26	31.01	30.66
135	**bta-miR-4523**	33.33	35.98	34.19	34.65	34.11	34.44	31.76	34.67	34.82	35.06	33.05	33.92
136	**bta-miR-453**	33.11	35.81	32.87	33.71	35.16	.	32.60	32.64	33.55	34.16	33.88	35.03
137	**bta-miR-433**	30.15	30.38	30.02	28.69	29.31	29.83	29.60	30.53	30.65	28.42	28.34	28.78
138	**bta-miR-449a**	31.16	33.54	32.61	32.39	32.56	30.72	28.57	33.34	31.93	36.04	.	.
139	**bta-miR-449b**	31.90	34.35	33.82	.	32.85	32.45	29.35	33.90	33.32	.	.	.
140	**bta-miR-484**	32.22	33.92	.	32.98	.	33.13	29.69	34.85	34.23	.	.	.
141	**bta-miR-485**	33.74	35.24	35.99	35.14	36.10	33.98	32.06	35.78	34.43	.	35.88	.
142	**bta-miR-486**	27.18	27.53	28.80	27.29	29.00	33.40	.	29.06	25.74	34.97	35.37	30.91
143	**bta-miR-500**	.	.	.	36.04	34.79	33.90	31.24	34.79	33.29	.	.	.

144	**bta-miR-502b**	33.97	36.76	36.47	34.83	.	33.38	33.49	.	.	.	31.77	32.34
145	**bta-miR-489**	31.89	31.82	30.98	29.99	31.20	32.31	30.54	32.08	31.16	30.55	29.84	31.90
146	**bta-miR-503-3p**	30.49	30.84	32.76	30.85	31.63	32.38	28.13	31.52	30.63	34.94	33.46	33.88
147	**bta-miR-493**	27.40	28.74	29.40	28.39	30.21	33.87	25.43	29.80	28.24	.	33.97	31.82
148	**bta-miR-505**	28.81	29.81	29.87	29.23	29.81	31.72	25.80	30.84	29.32	.	34.33	32.66
149	**bta-miR-494**	17.75	19.24	20.42	18.33	18.96	21.71	15.30	19.62	18.11	.	24.98	22.64
150	**bta-miR-541**	30.80	30.73	30.61	30.13	30.82	31.97	29.66	30.82	30.69	31.71	31.39	31.60
151	**bta-miR-584**	31.86	31.79	32.89	31.94	33.10	34.60	28.17	33.91	.	.	.	35.05
152	**bta-miR-592**	33.43	33.90	35.16	36.31	.	34.52	33.53	36.97	36.58	.	.	.
153	**bta-miR-615**	9.78	9.69	9.52	9.72	10.76	11.84	9.66	9.79	9.71	9.34	9.68	10.79
154	**bta-miR-631**	19.50	19.58	19.75	18.91	18.74	18.48	19.23	19.38	19.54	20.16	18.80	18.79
155	**bta-miR-574**	27.85	29.37	29.20	30.11	29.65	29.65	25.82	30.35	28.71	32.94	33.92	31.85
156	**bta-miR-656**	35.98	30.47	31.23	30.80	32.09	33.19	.	.	.	33.45	32.02	32.84
157	**bta-miR-664a**	32.00	32.83	32.99	33.76	34.04	35.87	29.75	.	32.99	.	.	36.43
158	**bta-miR-760-3p**	33.93	32.70	33.89	34.40	33.32	.	29.84	.	32.78	.	.	33.94
159	**bta-miR-664b**	33.96	35.77	35.20	35.84	34.37	.	30.79	34.18	32.85	.	34.78	.
160	**bta-miR-760-5p**	30.66	31.66	32.00	31.47	31.78	33.64	27.70	32.54	30.46	.	35.33	33.38
161	**bta-miR-669**	30.96	31.51	32.46	33.08	31.86	33.46	27.85	33.00	30.54	.	34.22	34.57
162	**bta-miR-763**	32.78	32.64	32.96	32.71	32.62	36.98	29.37	33.87	32.98	.	32.90	33.37
163	**bta-miR-764**	33.88	35.06	35.82	34.22	.	36.31	30.37	36.06	34.90	.	.	.
164	**bta-miR-767**	31.44	31.57	31.65	31.67	31.87	33.06	30.64	31.51	31.00	33.37	33.05	33.64
165	**bta-miR-677**	32.61	32.63	35.00	34.74	34.80	35.90	28.70	34.98	32.92	.	.	.
166	**bta-miR-874**	30.77	32.29	32.84	32.83	32.06	32.96	28.05	32.84	30.64	35.69	.	33.10
167	**bta-miR-877**	29.76	31.05	31.62	30.92	31.72	32.83	27.66	31.34	29.67	.	35.09	32.71
168	**bta-miR-885**	31.87	34.58	32.85	32.88	.	33.98	28.82	33.98	.	.	.	35.39
169	**bta-miR-99a-5p**	29.59	34.15	31.55	31.45	31.68	30.82	27.99	32.09	30.88	.	33.95	.
170	**bta-miR-9-5p**	31.87	.	33.95	.	33.69	33.11	32.93	36.66	33.22	35.76	.	33.12
171	**bta-miR-92a**	28.29	30.66	30.76	31.12	30.61	30.58	26.60	32.16	30.70	.	34.01	32.84
172	**bta-miR-92b**	26.80	27.76	28.68	28.07	27.78	28.33	24.77	28.56	26.99	32.43	31.80	30.50
173	**bta-miR-93**	30.68	34.94	33.20	33.89	31.73	30.92	29.32	33.15	33.87	35.10	31.73	30.71
174	**bta-miR-935**	32.86	32.35	32.58	33.27	32.76	32.04	29.50	.	32.55	.	36.86	35.38
175	**bta-miR-940**	25.36	23.46	26.83	26.52	27.82	29.49	22.83	25.68	23.70	31.65	30.70	29.73
176	**bta-miR-1224**	23.68	24.33	24.73	24.78	25.50	27.81	20.38	26.13	23.74	30.24	28.71	26.75
177	**bta-miR-1225-3p**	26.71	25.25	28.41	28.20	29.05	30.18	24.46	27.41	25.57	34.23	32.53	31.56
178	**bta-miR-1246**	21.75	24.94	22.63	.	24.87	24.53	20.44	.	22.42	.	28.64	28.01
179	**bta-miR-1247-3p**	29.76	31.60	32.19	30.85	31.79	31.87	27.57	31.89	30.46	35.04	33.32	33.99
180	**bta-miR-1296**	32.02	32.17	36.57	33.70	34.38	36.03	28.67	33.92	31.35	.	.	.
181	**bta-miR-1247-5p**	26.73	27.30	29.07	28.18	28.74	30.64	23.81	28.81	26.73	.	32.93	30.88
182	**bta-miR-1248**	34.26	32.88	34.73	33.96	35.66	.	29.41	.	33.86	.	33.98	.
183	**bta-miR-1301**	.	36.10	.	33.51	33.55	32.20	33.87	.	.	36.00	35.03	.
184	**bta-miR-1249**	32.88	32.85	33.98	34.45	31.76	31.18	29.42	36.12	32.14	.	32.50	32.86
185	**bta-miR-1260b**	22.75	25.71	24.16	24.77	24.41	26.83	20.84	25.64	23.93	30.83	28.62	26.83
186	**bta-miR-1307**	23.79	24.22	26.04	24.41	25.41	27.70	20.82	25.54	23.60	32.83	25.60	27.75
187	**bta-miR-1343-3p**	29.69	31.45	31.84	31.97	30.84	31.42	27.66	31.72	30.68	.	26.34	32.77
188	**bta-miR-1343-5p**	25.09	24.71	27.04	26.36	27.71	30.23	22.45	26.82	24.32	34.38	.	29.43
189	**bta-miR-1281**	30.82	29.70	31.03	31.45	30.78	30.82	27.27	31.21	29.36	.	22.37	31.76
190	**bta-miR-1388-3p**	32.26	31.69	31.80	31.81	32.22	34.14	28.10	32.81	31.27	.	28.51	36.39
191	**bta-miR-1287**	33.20	34.87	34.55	35.44	.	36.06	32.88	.	33.07	.	35.04	35.11
192	**bta-miR-1291**	33.97	32.88	.	.	32.93	.	32.67	.	34.15	.	30.92	.
