# Supplementary material for: Changes in Oviductal Cells and Small Extracellular Vesicles miRNAs in Pregnant Cows
Source: Front Vet Sci. 2021 Mar 4;8:639752. doi: 10.3389/fvets.2021.639752 (PMC7969882; doi:10.3389/fvets.2021.639752)
Supplement: Supplementary file 3 [file Table_2.pdf]

S2 Table. Full list of biological pathways with significant p-value ($p<0.05$) predicted as modulated by the 8 miRNAs (bta-miR-126-5p, bta-miR-129, bta-miR-140, bta-miR-188, bta-miR-219, bta-miR-345-3p, bta-miR-4523 and bta-miR-760-3p) up-regulated in OF-sEVs from pregnant compared to non-pregnant cows.

Pathways	Number of genes	P-value
bta00564 Glycerophospholipid metabolism	57	0.0003
bta01100 Metabolic pathways	552	0.0005
bta04070 Phosphatidylinositol signaling system	53	0.0008
bta04514 Cell adhesion molecules (CAMs)	75	0.0012
bta04012 ErbB signaling pathway	46	0.0012
bta04144 Endocytosis	106	0.0017
bta04152 AMPK signaling pathway	60	0.0021
bta04014 Ras signaling pathway	104	0.0022
bta05200 Pathways in cancer	209	0.0024
bta04928 Parathyroid hormone synthesis, secretion and action	52	0.0027
bta04071 Sphingolipid signaling pathway	58	0.0028
bta04921 Oxytocin signaling pathway	70	0.0029
bta04931 Insulin resistance	54	0.003
bta04659 Th17 cell differentiation	55	0.0032
bta04910 Insulin signaling pathway	65	0.0035
bta00562 Inositol phosphate metabolism	39	0.0036
bta04150 mTOR signaling pathway	71	0.0039
bta04072 Phospholipase D signaling pathway	69	0.0041
bta04810 Regulation of actin cytoskeleton	90	0.0048
bta04360 Axon guidance	78	0.0048
bta01521 EGFR tyrosine kinase inhibitor resistance	41	0.0051
bta05017 Spinocerebellar ataxia	47	0.0055
bta04010 MAPK signaling pathway	118	0.0058
bta05205 Proteoglycans in cancer	87	0.006
bta05214 Glioma	39	0.0071
bta04722 Neurotrophin signaling pathway	56	0.0074
bta04270 Vascular smooth muscle contraction	60	0.0076
bta04961 Endocrine and other factor-regulated calcium reabsorption	28	0.0078
bta04261 Adrenergic signaling in cardiomyocytes	66	0.0082
bta04066 HIF-1 signaling pathway	51	0.0088
bta04310 Wnt signaling pathway	70	0.0091
bta05211 Renal cell carcinoma	36	0.0092
bta04142 Lysosome	59	0.0093
bta04530 Tight junction	75	0.0102
bta05231 Choline metabolism in cancer	46	0.012
bta04130 SNARE interactions in vesicular transport	20	0.0124
bta05100 Bacterial invasion of epithelial cells	36	0.0126
bta04020 Calcium signaling pathway	83	0.0129
bta04062 Chemokine signaling pathway	78	0.013
bta05220 Chronic myeloid leukemia	37	0.0154
bta00760 Nicotinate and nicotinamide metabolism	22	0.0161
bta04670 Leukocyte transendothelial migration	50	0.0176
bta04721 Synaptic vesicle cycle	37	0.0178
bta04022 cGMP-PKG signaling pathway	70	0.0181
bta04510 Focal adhesion	80	0.0191
bta04750 Inflammatory mediator regulation of TRP channels	46	0.0196
bta04216 Ferroptosis	24	0.0196

bta04658 Th1 and Th2 cell differentiation	44	0.0207
bta04666 Fc gamma R-mediated phagocytosis	42	0.0218
bta04919 Thyroid hormone signaling pathway	51	0.0226
bta05167 Kaposi sarcoma-associated herpesvirus infection	82	0.023
bta01522 Endocrine resistance	42	0.0246
bta05135 Yersinia infection	55	0.0247
bta05321 Inflammatory bowel disease (IBD)	33	0.0255
bta01230 Biosynthesis of amino acids	34	0.0267
bta04922 Glucagon signaling pathway	45	0.0269
bta04151 PI3K-Akt signaling pathway	138	0.027
bta05202 Transcriptional misregulation in cancer	76	0.0276
bta05212 Pancreatic cancer	35	0.0278
bta04120 Ubiquitin mediated proteolysis	58	0.0287
bta04371 Apelin signaling pathway	58	0.0287
bta04211 Longevity regulating pathway	40	0.0293
bta04015 Rap1 signaling pathway	84	0.0318
bta05170 Human immunodeficiency virus 1 infection	90	0.0328
bta04024 cAMP signaling pathway	88	0.0349
bta04728 Dopaminergic synapse	55	0.0364
bta00230 Purine metabolism	55	0.0364
bta04664 Fc epsilon RI signaling pathway	32	0.0365
bta05163 Human cytomegalovirus infection	93	0.0382
bta05215 Prostate cancer	42	0.0387
bta04668 TNF signaling pathway	49	0.0401
bta04730 Long-term depression	28	0.0405
bta04611 Platelet activation	50	0.0407
bta04660 T cell receptor signaling pathway	45	0.0412
bta05235 PD-L1 expression and PD-1 checkpoint pathway in cancer	40	0.0412
bta04925 Aldosterone synthesis and secretion	41	0.0422
bta05230 Central carbon metabolism in cancer	30	0.0439
bta04370 VEGF signaling pathway	27	0.0445
bta04924 Renin secretion	32	0.0469
bta04725 Cholinergic synapse	47	0.0471
