# Supplementary material for: Changes in Oviductal Cells and Small Extracellular Vesicles miRNAs in Pregnant Cows
Source: Front Vet Sci. 2021 Mar 4;8:639752. doi: 10.3389/fvets.2021.639752 (PMC7969882; doi:10.3389/fvets.2021.639752)
Supplement: Supplementary file 4 [file Table_3.pdf]

S3 Table. Full list of biological pathways with significant p-value ($p<0.05$) predicted as modulated by the miRNAs (bta-miR-331-5p) up-regulated in OF-sEVs from non-pregnant compared to pregnant cows.

Pathways	Number of genes	P-value
bta05211 Renal cell carcinoma	12	0
bta04919 Thyroid hormone signaling pathway	13	0.0003
bta04910 Insulin signaling pathway	13	0.0012
bta04931 Insulin resistance	11	0.0016
bta04012 ErbB signaling pathway	9	0.0027
bta04360 Axon guidance	14	0.0034
bta04662 B cell receptor signaling pathway	9	0.0034
bta05210 Colorectal cancer	9	0.0039
bta04370 VEGF signaling pathway	7	0.0043
bta05163 Human cytomegalovirus infection	17	0.0046
bta05212 Pancreatic cancer	8	0.005
bta04022 cGMP-PKG signaling pathway	13	0.0055
bta05200 Pathways in cancer	30	0.0061
bta04922 Glucagon signaling pathway	9	0.0091
bta05221 Acute myeloid leukemia	7	0.0093
bta04140 Autophagy	11	0.0095
bta00510 N-Glycan biosynthesis	6	0.0096
bta00564 Glycerophospholipid metabolism	9	0.0096
bta04310 Wnt signaling pathway	12	0.0096
bta04660 T cell receptor signaling pathway	9	0.0113
bta04024 cAMP signaling pathway	15	0.0115
bta04512 ECM-receptor interaction	8	0.0116
bta04066 HIF-1 signaling pathway	9	0.0132
bta05170 Human immunodeficiency virus 1 infection	15	0.0136
bta04072 Phospholipase D signaling pathway	11	0.0148
bta05225 Hepatocellular carcinoma	12	0.0156
bta04728 Dopaminergic synapse	10	0.0162
bta05213 Endometrial cancer	6	0.0163
bta05205 Proteoglycans in cancer	13	0.0218
bta04550 Signaling pathways regulating pluripotency of stem cells	10	0.0226
bta04152 AMPK signaling pathway	9	0.0241
bta04810 Regulation of actin cytoskeleton	13	0.0264
bta04921 Oxytocin signaling pathway	10	0.033
bta05226 Gastric cancer	10	0.0342
bta04510 Focal adhesion	12	0.0352
bta04380 Osteoclast differentiation	9	0.0375
bta04670 Leukocyte transendothelial migration	8	0.0376
bta04390 Hippo signaling pathway	10	0.038
bta05235 PD-L1 expression and PD-1 checkpoint pathway in cancer	7	0.0384
bta04514 Cell adhesion molecules (CAMs)	10	0.0406
bta05165 Human papillomavirus infection	18	0.0416
bta05412 Arrhythmogenic right ventricular cardiomyopathy (ARVC)	6	0.0443
bta05220 Chronic myeloid leukemia	6	0.0465
bta05231 Choline metabolism in cancer	7	0.0499
