# Supplementary material for: Changes in Oviductal Cells and Small Extracellular Vesicles miRNAs in Pregnant Cows
Source: Front Vet Sci. 2021 Mar 4;8:639752. doi: 10.3389/fvets.2021.639752 (PMC7969882; doi:10.3389/fvets.2021.639752)
Supplement: Supplementary file 5 [file Table_4.pdf]

S4 Table. Cycle threshold levels of the 363 miRNAs detected in ovidutal epithelial cells from non-pregnant (OEC/Non-pregnant) and pregnant (OEC/Pregnant) cows.

	MicroRNAs	Group											
		OEC/Non-pregnant						OEC/Pregnant					
		1	2	3	4	5	6	1	2	3	4	5	6
**(n)**	**Detected in OEC/Non-pregnant**												
1	**bta-miR-124b**	29.83	.	29.83	31.70	.	29.65	29.83	.	.	.	.	29.72
2	**bta-miR-217**	34.98	.	.	36.33	33.78	33.89	.	34.43	.	.	.	33.71
3	**bta-miR-329a**	36.04	34.90	.	.	.	35.89	33.97	34.39	.	.	.	.
4	**bta-miR-377**	36.04	.	.	35.73	35.91	36.76	.	.	.	.	.	35.39
5	**bta-miR-411c-5p**	33.56	.	.	34.59	.	34.39	35.70	.	.	36.75	.	.
6	**bta-miR-487a**	29.73	32.35	.	31.61	.	.	.	30.81	.	.	.	.
7	**bta-miR-539**	32.60	35.01	33.00	33.94	31.23	34.16	.	.	.	.	35.57	33.98
8	**bta-miR-655**	32.90	.	34.42	34.34	.	36.25	33.17	.	.	.	.	36.02
9	**bta-miR-875**	36.95	.	35.13	.	33.02	33.91	35.00	35.71	.	.	.	.
10	**bta-miR-9-3p**	34.57	36.41	.	.	34.75	36.85	.	.	.	.	.	35.04
**(n)**	**Detected in OEC/Pregnant**												
1	**bta-miR-127**	.	.	.	.	.	26.58	25,79	25,63	.	.	.	25,83
2	**bta-miR-302b**	35.99	35.21	.	.	.	.	.	34,49	.	.	35,80	35,37
3	**bta-miR-331-5p**	.	.	27.92	.	.	.	.	.	.	29,12	29,45	27,92
4	**bta-miR-4523**	.	.	28.66	.	.	.	.	.	.	30,03	30,57	28,60
5	**bta-miR-504**	.	.	27.65	27.28	.	.	.	29,77	27,79	27,59	.	26,77
6	**bta-miR-542-5p**	.	.	33.71	.	.	.	.	.	32,96	33,81	36,61	32,43
7	**bta-miR-544a**	31.55	.	32.82	.	.	.	32,39	32,51	.	34,10	35,73	33,14
8	**bta-miR-1197**	32.95	.	33.70	.	.	.	33,91	35,38	35,05	33,54	34,97	33,18
**(n)**	**Detected in OEC/Non-pregnant and OEC/Pregnant**												
1	**bta-let-7a-3p**	26.37	27.86	27.32	25.75	23.80	25.67	25.75	25.73	26.06	26.03	26.88	25.60
2	**bta-miR-103**	24.76	26.75	25.47	24.08	22.66	24.14	25.09	24.81	25.09	24.81	26.12	23.75
3	**bta-let-7a-5p**	18.15	20.05	18.24	18.73	17.66	18.66	18.50	18.33	19.42	18.77	20.41	18.20
4	**bta-miR-105a**	30.11	31.48	30.78	31.77	28.45	31.44	.	.	32.94	31.29	.	30.47
5	**bta-let-7b**	16.75	18.20	16.84	17.43	16.55	17.66	16.93	16.74	18.00	17.27	18.56	17.30
6	**bta-miR-105b**	28.74	30.34	29.76	28.79	25.68	28.62	29.05	28.53	29.26	29.35	29.54	28.70
7	**bta-let-7c**	16.96	18.67	16.89	17.63	16.49	17.77	17.27	17.01	18.00	17.69	18.83	17.35
8	**bta-miR-106a**	25.09	27.34	25.88	23.80	22.56	23.41	24.98	25.40	24.67	25.20	25.33	23.00
9	**bta-let-7d**	19.42	21.14	19.49	19.84	18.80	20.04	19.58	19.74	21.03	20.13	21.74	19.55
10	**bta-miR-106b**	.	28.36	26.83	25.45	23.81	25.79	26.40	26.76	26.57	26.88	27.14	25.29
11	**bta-let-7e**	17.57	19.43	17.51	18.00	16.76	18.67	17.79	17.83	18.89	18.12	19.74	17.57
12	**bta-miR-107**	28.33	29.84	29.55	29.01	26.80	28.80	29.00	28.43	29.74	29.80	30.48	28.78
13	**bta-let-7f**	20.73	22.85	21.08	21.41	19.99	21.13	20.79	21.22	22.10	21.63	22.92	20.64
14	**bta-miR-10a**	24.08	26.01	24.77	23.45	22.78	22.71	24.74	24.61	24.44	24.35	25.84	22.58
15	**bta-let-7g**	22.90	24.70	23.21	22.92	21.60	22.79	23.01	23.07	24.42	23.34	24.75	22.24
16	**bta-miR-10b**	24.75	26.69	25.50	23.87	23.10	23.46	25.34	25.48	24.85	24.88	26.35	22.83
17	**bta-let-7i**	23.78	25.69	24.40	24.54	22.10	24.21	23.83	24.27	24.75	24.33	25.66	24.29
18	**bta-miR-1**	31.20	33.36	31.74	32.02	29.88	30.92	32.28	.	31.84	32.15	32.67	30.51
19	**bta-miR-124a**	.	31.00	30.74	33.87	.	30.47	.	.	31.80	30.66	.	30.25
20	**bta-miR-100**	23.11	24.69	23.78	21.46	20.83	21.46	22.50	22.35	22.69	22.33	22.99	21.57
21	**bta-miR-101**	27.51	29.98	27.89	25.40	23.33	25.82	27.61	28.09	27.07	27.89	27.76	24.90
22	**bta-miR-125a**	22.01	22.84	22.12	21.65	20.59	21.40	21.82	22.05	22.78	21.70	23.84	21.64
23	**bta-miR-125b**	20.30	21.35	20.51	19.73	18.79	20.12	19.83	19.85	21.53	20.31	21.81	20.06
24	**bta-miR-133b**	32.31	32.91	.	32.92	.	31.87	32.82	32.48	35.24	34.53	34.14	32.99
25	**bta-miR-126-3p**	28.80	31.43	30.49	27.45	27.13	28.75	28.82	31.83	28.96	28.75	30.75	27.72

26	**bta-miR-133c**	31.91	35.81	32.88	.	32.66	33.32	31.78	32.77	35.95	.	.	34.82
27	**bta-miR-126-5p**	27.68	29.95	28.84	27.49	27.37	29.05	27.76	29.83	29.31	28.60	30.32	28.07
28	**bta-miR-134**	26.94	28.78	26.90	28.07	26.19	27.05	27.48	27.74	28.74	28.81	.	25.80
29	**bta-miR-135a**	22.83	24.10	23.77	21.67	20.02	21.54	21.81	22.08	22.00	21.75	22.26	21.89
30	**bta-miR-128**	25.93	27.55	26.06	25.82	24.56	25.81	25.78	25.82	26.62	25.78	27.81	25.57
31	**bta-miR-135b**	24.19	25.70	25.52	22.96	21.68	22.94	23.03	23.62	23.58	23.62	23.72	23.57
32	**bta-miR-129**	26.75	27.93	26.87	27.71	25.68	27.16	26.74	27.33	27.89	28.00	28.73	26.80
33	**bta-miR-136**	31.28	32.64	32.78	33.23	31.22	32.80	31.81	30.88	33.82	32.36	34.30	31.94
34	**bta-miR-129-3p**	29.02	30.81	30.57	29.74	28.68	29.77	29.77	30.33	30.44	29.63	31.87	29.39
35	**bta-miR-137**	34.78	.	36.83	36.34	30.72	35.21	36.43	35.00	33.49	32.90	34.02	.
36	**bta-miR-129-5p**	26.68	27.99	27.16	27.78	25.73	26.93	26.94	.	27.81	27.85	28.68	27.02
37	**bta-miR-138**	25.34	27.84	25.89	.	23.71	26.68	25.89	25.68	26.61	.	27.43	26.22
38	**bta-miR-130a**	29.32	30.40	29.68	29.21	26.77	28.70	29.17	29.57	28.98	28.83	30.72	29.47
39	**bta-miR-139**	27.96	28.93	27.78	27.80	26.18	27.49	26.62	27.45	28.02	27.95	28.79	27.75
40	**bta-miR-130b**	20.25	20.81	20.73	20.72	19.85	20.69	20.49	20.11	21.46	20.78	21.75	20.46
41	**bta-miR-140**	27.46	28.96	27.78	27.18	25.63	26.87	27.54	27.68	28.25	27.34	29.22	26.64
42	**bta-miR-132**	27.55	28.19	27.14	28.49	27.03	27.79	27.78	27.81	28.68	27.59	28.24	27.36
43	**bta-miR-141**	24.23	26.79	25.65	21.81	19.48	22.05	24.33	24.57	22.74	23.83	23.33	21.15
44	**bta-miR-133a**	27.75	.	27.80	.	25.72	27.99	28.23	27.84	.	28.78	.	28.08
45	**bta-miR-142-3p**	30.75	34.72	35.57	29.38	27.01	27.88	30.80	34.04	29.40	30.40	30.79	27.90
46	**bta-miR-142-5p**	30.36	.	31.86	31.70	28.80	31.02	30.60	31.82	31.77	31.43	34.20	30.28
47	**bta-miR-151-3p**	23.83	25.27	24.49	23.71	22.79	23.46	24.16	.	24.36	23.95	25.32	23.55
48	**bta-miR-143**	24.36	26.14	25.06	24.69	22.89	24.71	24.69	24.53	25.76	25.05	26.85	24.66
49	**bta-miR-151-5p**	21.81	23.52	22.45	21.97	20.76	21.86	22.10	21.93	22.73	22.46	23.78	21.86
50	**bta-miR-152**	.	31.88	29.08	30.52	27.45	28.93	.	29.70	.	31.53	31.81	29.39
51	**bta-miR-145**	25.80	.	26.63	25.84	23.32	26.61	26.59	26.01	25.84	25.62	.	26.47
52	**bta-miR-153**	29.70	31.72	31.37	27.11	24.82	26.70	28.84	29.26	26.21	27.30	26.67	26.84
53	**bta-miR-146a**	29.38	33.49	29.75	31.79	28.43	31.43	31.56	30.28	28.94	29.59	33.95	29.40
54	**bta-miR-154a**	30.10	30.47	31.06	30.68	.	30.58	30.66	29.80	31.36	30.90	31.88	30.70
55	**bta-miR-146b**	29.39	31.82	29.52	31.73	27.81	30.82	30.32	29.59	28.80	29.10	32.82	.
56	**bta-miR-154b**	31.58	30.29	29.74	31.13	30.13	30.54	31.68	30.77	31.83	30.90	30.77	30.08
57	**bta-miR-147**	27.47	29.71	28.75	29.46	27.38	29.22	27.80	28.52	28.72	30.49	31.49	28.69
58	**bta-miR-154c**	33.53	32.80	33.52	33.06	31.78	32.85	33.10	32.95	34.01	34.96	33.43	33.09
59	**bta-miR-148a**	21.98	23.87	22.35	21.04	19.27	21.52	21.74	22.18	22.58	22.34	22.74	21.67
60	**bta-miR-155**	26.77	28.20	27.30	27.02	27.52	27.81	27.22	27.30	27.76	27.72	28.76	26.56
61	**bta-miR-148b**	22.65	24.62	22.77	21.64	19.52	22.00	22.42	22.55	22.92	22.80	23.17	21.77
62	**bta-miR-15a**	25.15	27.14	25.69	24.51	22.77	24.12	25.35	24.88	25.18	24.98	25.68	23.80
63	**bta-miR-15b**	23.02	24.40	23.47	23.61	23.07	23.51	23.27	22.97	23.89	23.24	24.74	23.18
64	**bta-miR-149-5p**	26.71	28.42	.	27.92	25.54	27.46	26.81	27.22	28.10	27.74	28.73	26.79
65	**bta-miR-16a**	22.73	24.69	23.68	22.25	21.17	22.06	22.76	22.72	23.20	22.73	23.81	21.87
66	**bta-miR-150**	27.06	28.44	28.43	27.75	26.60	26.79	27.65	27.98	28.24	27.37	30.27	26.82
67	**bta-miR-16b**	21.70	23.73	22.76	21.74	20.65	21.50	21.84	21.83	22.50	22.01	22.99	21.47
68	**bta-miR-17-3p**	28.75	30.74	.	.	27.17	28.97	28.68	28.74	30.02	30.08	29.94	28.58
69	**bta-miR-188**	27.71	29.23	28.56	29.63	25.77	28.38	28.88	28.55	.	.	29.52	28.74
70	**bta-miR-17-5p**	27.67	29.65	28.78	26.86	25.07	26.24	27.73	27.95	27.43	27.99	28.25	26.21
71	**bta-miR-18a**	30.28	31.83	31.70	29.66	27.71	28.74	29.94	.	29.85	29.70	30.49	28.05
72	**bta-miR-181a**	28.48	29.65	29.33	27.06	25.31	26.77	28.30	28.38	27.77	27.83	28.73	27.43
73	**bta-miR-18b**	.	34.32	31.88	30.91	29.08	30.60	31.88	31.68	30.78	32.49	32.11	29.78
74	**bta-miR-181b**	26.29	27.97	26.77	26.46	24.75	26.46	26.72	26.54	26.78	26.28	27.80	26.76
75	**bta-miR-190a**	34.14	.	34.28	29.38	28.69	29.19	31.62	32.72	31.65	31.16	31.81	29.37
76	**bta-miR-181c**	29.55	30.80	30.00	28.75	26.55	28.29	29.77	29.54	.	29.34	29.76	28.50
77	**bta-miR-190b**	25.18	26.83	25.90	25.29	23.84	24.59	25.70	25.46	25.94	25.20	25.74	24.51
78	**bta-miR-181d**	24.96	26.92	25.59	25.57	24.42	25.72	25.52	25.44	25.96	25.14	26.46	25.80
79	**bta-miR-191**	22.67	23.53	22.81	22.70	21.66	22.68	22.60	22.60	23.80	22.80	24.47	22.38
80	**bta-miR-182**	27.47	29.14	27.91	27.23	25.84	27.00	27.19	27.76	28.01	27.64	28.62	27.26

81	**bta-miR-192**	28.61	29.75	.	28.69	26.69	28.73	28.78	.	30.01	29.43	30.66	28.32
82	**bta-miR-183**	27.13	29.18	27.81	27.73	26.83	27.76	27.39	27.81	28.44	27.77	29.17	27.79
83	**bta-miR-193a**	32.36	33.45	32.48	.	.	32.35	32.69	32.67	.	33.98	.	31.43
84	**bta-miR-184**	32.97	34.50	31.66	33.90	.	.	31.09	32.70	33.02	33.89	34.12	32.32
85	**bta-miR-193a-3p**	.	31.53	.	30.01	.	29.06	29.87	.	.	29.80	32.04	29.58
86	**bta-miR-185**	26.63	29.11	26.84	26.64	25.49	26.73	26.99	26.70	27.80	27.49	28.90	26.22
87	**bta-miR-193a-5p**	25.77	26.14	25.73	25.82	24.64	25.78	25.81	25.77	26.49	25.82	26.71	25.70
88	**bta-miR-186**	24.93	.	25.44	24.49	22.42	24.53	25.28	25.02	25.70	25.11	26.70	23.99
89	**bta-miR-187**	26.64	.	27.10	28.97	24.46	27.28	26.81	27.41	27.82	27.95	28.17	27.25
90	**bta-miR-194**	27.69	29.54	28.68	26.23	25.42	26.24	27.86	28.50	27.86	27.81	28.13	25.99
91	**bta-miR-195**	24.19	26.23	24.80	24.31	22.88	24.32	24.25	24.05	24.91	24.64	25.74	24.03
92	**bta-miR-200c**	18.73	19.84	18.82	18.62	18.64	18.76	18.80	18.80	19.41	18.72	20.12	18.44
93	**bta-miR-196a**	30.19	32.63	30.54	30.81	29.80	30.69	32.05	31.35	29.52	31.03	31.89	30.68
94	**bta-miR-202**	.	.	33.86	34.80	30.34	.	34.93	.	33.88	.	34.51	33.39
95	**bta-miR-196b**	.	33.79	31.87	30.08	29.74	30.70	32.83	.	29.60	31.88	32.94	31.42
96	**bta-miR-204**	.	26.66	25.88	26.49	23.79	27.01	24.79	25.05	26.72	25.73	26.64	23.64
97	**bta-miR-197**	23.71	24.59	23.97	24.75	23.49	24.60	24.53	24.05	25.06	24.49	25.77	23.84
98	**bta-miR-205**	23.82	25.60	23.84	24.40	23.28	23.91	25.21	25.74	24.76	24.72	27.04	24.52
99	**bta-miR-199a-3p**	28.51	33.10	28.78	27.96	26.72	28.68	29.79	30.61	29.14	28.25	31.03	28.26
100	**bta-miR-206**	27.83	29.77	.	29.37	26.68	28.66	28.50	28.12	29.37	.	30.31	28.58
101	**bta-miR-199a-5p**	29.35	33.82	.	30.30	27.78	30.71	30.84	31.37	30.38	29.50	32.59	30.71
102	**bta-miR-208a**	31.58	33.04	31.89	33.34	30.80	32.58	.	31.92	34.12	33.65	33.88	32.31
103	**bta-miR-199b**	29.78	34.45	30.65	29.91	28.15	30.71	31.06	32.07	31.56	29.53	32.19	30.30
104	**bta-miR-208b**	32.92	32.88	33.28	32.67	31.78	33.00	32.87	32.88	32.67	33.13	34.46	32.34
105	**bta-miR-199c**	27.76	30.51	28.36	26.94	25.83	27.75	28.69	29.52	28.03	26.98	30.36	27.06
106	**bta-miR-20a**	25.08	27.14	25.99	23.70	22.67	23.18	25.10	25.46	24.58	24.90	25.03	23.10
107	**bta-miR-19a**	27.67	30.72	28.61	27.55	25.70	27.36	28.25	28.97	.	28.99	29.83	27.09
108	**bta-miR-20b**	26.44	28.79	27.28	25.13	23.70	24.67	26.65	26.72	25.95	26.67	26.74	24.71
109	**bta-miR-19b**	27.82	30.59	28.63	27.35	25.49	27.07	28.51	28.79	.	28.50	29.43	26.74
110	**bta-miR-21-3p**	.	30.65	29.48	30.19	.	30.22	30.22	29.79	30.76	30.41	.	.
111	**bta-miR-200a**	24.99	27.28	25.78	23.13	21.06	23.71	25.29	25.08	24.68	24.84	25.76	22.89
112	**bta-miR-21-5p**	25.81	28.17	26.39	25.78	24.81	25.70	26.53	26.52	26.67	26.21	27.82	25.25
113	**bta-miR-200b**	17.52	19.07	17.81	17.16	16.73	17.22	17.70	17.53	18.31	17.47	19.03	16.72
114	**bta-miR-210**	25.04	27.29	25.69	26.44	24.56	25.97	26.15	25.33	27.59	26.38	27.88	25.85
115	**bta-miR-211**	25.92	27.50	25.80	26.81	24.40	27.36	25.51	25.77	27.76	26.50	27.57	24.62
116	**bta-miR-22-5p**	27.72	29.76	28.49	27.03	25.54	27.63	27.76	27.62	28.07	27.55	28.63	27.55
117	**bta-miR-212**	30.80	31.86	.	32.85	.	.	30.81	.	33.01	32.96	33.52	31.44
118	**bta-miR-221**	25.11	26.83	26.79	27.17	21.54	26.08	25.78	25.59	26.45	26.41	26.57	26.53
119	**bta-miR-214**	26.57	28.54	27.19	28.77	25.01	.	26.95	27.32	28.62	.	28.81	27.37
120	**bta-miR-222**	23.46	24.40	23.89	24.46	22.81	24.14	23.62	23.72	24.46	23.97	24.82	23.72
121	**bta-miR-215**	29.24	31.51	30.36	28.56	27.75	28.73	29.45	30.44	29.87	29.27	29.96	28.46
122	**bta-miR-223**	32.51	31.84	30.03	30.78	29.88	31.88	31.01	30.73	33.27	32.15	31.81	30.81
123	**bta-miR-216a**	28.14	29.99	28.69	29.77	26.86	29.59	28.16	28.31	29.70	29.52	29.15	29.15
124	**bta-miR-224**	26.79	27.93	27.30	26.91	25.15	26.72	26.80	26.76	27.74	26.68	28.55	27.32
125	**bta-miR-216b**	28.82	30.74	29.42	30.39	28.48	29.83	29.41	.	30.81	30.83	31.67	28.89
126	**bta-miR-23a**	19.40	20.82	19.72	19.12	18.81	19.49	19.80	19.56	20.21	19.49	20.85	18.93
127	**bta-miR-23b-3p**	22.19	23.81	22.58	22.27	21.67	22.23	22.60	22.63	22.99	22.63	23.79	21.80
128	**bta-miR-218**	30.28	32.63	30.75	29.92	27.85	30.09	30.71	30.18	30.17	29.64	31.62	29.87
129	**bta-miR-23b-5p**	.	31.09	29.43	30.08	27.72	30.09	.	29.67	29.76	31.82	30.64	29.59
130	**bta-miR-24**	31.71	35.64	33.16	30.08	28.16	30.38	32.32	32.05	29.96	32.84	31.62	.
131	**bta-miR-219-3p**	.	.	27.69	28.41	26.11	28.04	27.68	.	.	28.41	28.70	27.71
132	**bta-miR-24-3p**	22.81	24.61	22.87	21.67	20.52	21.49	22.71	23.30	22.76	22.55	24.03	21.25
133	**bta-miR-219-5p**	35.41	.	.	35.74	31.82	.	34.07	.	.	34.47	33.94	34.19
134	**bta-miR-25**	21.66	23.12	22.16	21.70	21.35	21.42	22.09	21.85	22.33	21.69	23.26	21.32
135	**bta-miR-22-3p**	2.92	3.05	3.07	2.82	2.49	3.17	2.84	2.92	3.03	2.89	2.93	2.84

136	**bta-miR-26a**	19.82	21.41	19.81	19.07	17.74	18.77	19.81	19.77	20.25	19.72	20.85	18.79
137	**bta-miR-26b**	22.65	24.54	22.77	21.70	20.36	21.54	22.46	22.81	22.88	22.46	23.66	21.29
138	**bta-miR-29d-3p**	24.23	25.82	24.75	22.76	20.81	22.73	23.58	24.13	23.92	24.00	24.06	22.48
139	**bta-miR-26c**	32.86	.	33.82	34.92	31.88	.	34.63	34.98	.	.	36.63	33.96
140	**bta-miR-29d-5p**	26.41	28.13	27.32	26.73	24.76	26.48	26.78	26.85	27.72	26.79	28.06	26.78
141	**bta-miR-27a-3p**	23.55	25.53	23.72	22.82	20.72	22.81	23.81	23.49	24.01	23.57	24.89	22.79
142	**bta-miR-29e**	29.66	35.74	30.51	26.80	24.55	27.41	29.18	30.59	28.82	29.62	28.63	26.58
143	**bta-miR-27a-5p**	29.62	.	29.73	29.52	27.64	29.35	29.64	.	31.05	29.72	30.28	29.10
144	**bta-miR-301a**	33.34	33.86	.	32.81	30.04	33.63	33.56	31.84	32.91	33.34	.	32.16
145	**bta-miR-27b**	23.24	24.98	23.34	22.66	21.25	22.48	23.70	23.42	23.83	23.53	24.71	21.81
146	**bta-miR-301b**	32.34	33.14	33.80	32.64	.	33.04	31.88	33.37	33.50	32.32	33.83	31.80
147	**bta-miR-28**	27.17	28.80	27.56	25.88	25.46	25.62	27.48	27.43	27.24	27.30	28.18	25.73
148	**bta-miR-302a**	.	33.51	.	35.81	.	34.49	.	36.21	36.38	.	35.88	33.97
149	**bta-miR-296-3p**	25.96	27.62	26.62	27.31	24.72	26.74	.	26.61	27.32	27.25	27.70	26.45
150	**bta-miR-296-5p**	26.43	28.23	.	.	25.28	26.92	26.94	.	27.83	27.77	28.76	.
151	**bta-miR-302c**	31.87	34.99	31.90	32.84	30.82	32.06	32.13	32.76	35.29	33.98	32.88	32.27
152	**bta-miR-299**	34.65	34.89	32.92	.	32.21	.	35.84	35.25	35.49	33.37	.	36.23
153	**bta-miR-302d**	33.89	33.90	31.42	.	.	32.86	34.89	31.82	.	34.01	.	31.68
154	**bta-miR-29a**	20.53	22.00	20.41	19.22	18.24	19.32	19.94	20.57	20.84	20.22	21.39	19.32
155	**bta-miR-3064**	28.10	32.52	28.00	32.49	27.73	28.30	27.93	30.39	30.50	30.20	32.70	28.73
156	**bta-miR-29b**	26.15	29.79	27.27	24.80	21.93	25.04	26.55	26.99	26.19	26.47	26.71	24.58
157	**bta-miR-30a-5p**	24.65	26.23	25.39	23.80	21.86	23.39	24.69	24.76	25.18	24.77	25.82	23.43
158	**bta-miR-29c**	20.54	22.07	20.50	19.31	18.20	19.36	19.97	20.62	20.73	20.26	21.28	19.31
159	**bta-miR-30b-3p**	28.28	29.51	27.84	28.91	27.71	28.81	29.06	28.21	29.16	28.50	29.22	28.73
160	**bta-miR-30b-5p**	23.78	25.61	24.15	23.27	21.53	23.17	24.08	23.80	25.33	24.35	25.80	22.74
161	**bta-miR-328**	24.73	25.49	24.68	25.48	23.84	24.64	25.40	24.77	25.82	25.22	25.80	24.35
162	**bta-miR-30c**	22.70	23.69	23.01	22.78	20.94	22.19	23.20	22.76	23.97	23.24	24.83	21.76
163	**bta-miR-30d**	24.74	25.97	25.27	23.69	22.00	23.54	24.79	24.82	25.24	24.93	25.76	23.54
164	**bta-miR-329b**	31.29	35.72	.	34.77	30.61	31.59	32.10	32.93	33.50	36.72	35.99	31.84
165	**bta-miR-30e-5p**	24.63	26.22	25.29	23.83	21.89	23.54	24.84	24.86	25.23	24.90	26.10	23.55
166	**bta-miR-330**	.	29.62	27.33	.	25.81	27.73	27.76	.	.	28.93	29.66	27.79
167	**bta-miR-30f**	24.17	24.85	24.51	24.02	22.25	23.49	24.65	24.26	25.34	24.72	.	23.46
168	**bta-miR-331-3p**	26.59	26.73	25.79	24.73	24.23	24.66	25.80	26.80	25.36	24.84	27.34	25.16
169	**bta-miR-31**	22.91	25.46	23.26	22.69	21.11	22.22	23.58	23.32	24.09	23.36	25.13	21.63
170	**bta-miR-32**	31.86	32.82	33.81	30.69	28.48	31.02	32.26	31.77	29.89	.	31.68	30.90
171	**bta-miR-335**	30.78	35.29	32.95	32.62	29.46	32.62	31.88	33.51	31.90	33.70	30.87	31.55
172	**bta-miR-320a**	21.57	22.80	22.19	22.54	20.59	22.28	22.20	21.88	22.67	22.35	23.12	22.24
173	**bta-miR-338**	30.23	31.98	30.81	27.89	25.52	27.93	30.43	31.52	28.98	29.52	29.57	28.07
174	**bta-miR-320b**	26.80	28.70	.	28.60	24.35	27.87	27.39	27.04	27.84	28.31	27.84	27.76
175	**bta-miR-339a**	24.74	25.77	24.76	24.93	22.88	24.58	25.08	24.83	25.42	25.25	26.84	24.27
176	**bta-miR-323**	17.49	17.82	17.51	17.65	17.50	17.72	17.27	17.88	17.36	17.69	17.71	17.67
177	**bta-miR-339b**	23.77	24.81	23.59	23.87	21.78	23.47	24.04	23.79	24.67	24.13	25.83	23.48
178	**bta-miR-324**	28.50	29.12	28.79	27.49	.	26.89	28.17	28.82	28.19	27.81	.	26.67
179	**bta-miR-33a**	30.19	32.34	31.14	32.27	28.58	31.71	31.20	31.63	30.69	32.28	32.13	31.53
180	**bta-miR-326**	.	27.82	25.97	27.27	.	26.44	25.99	26.25	.	.	.	26.41
181	**bta-miR-33b**	30.80	32.91	31.81	32.45	29.97	32.03	.	31.08	32.29	33.30	34.65	30.73
182	**bta-miR-340**	32.54	33.16	31.13	30.62	29.64	29.72	30.73	30.99	31.54	30.73	31.92	30.86
183	**bta-miR-365-3p**	26.31	27.75	25.97	25.98	25.29	25.75	26.84	25.96	27.52	26.55	28.16	25.27
184	**bta-miR-342**	27.73	29.42	28.60	27.78	26.55	27.37	27.92	28.79	28.76	28.00	30.36	26.81
185	**bta-miR-365-5p**	26.60	.	25.99	28.92	25.06	26.06	26.92	28.09	29.71	.	29.68	26.69
186	**bta-miR-345-3p**	27.53	29.64	27.73	28.79	26.16	27.71	28.06	27.78	28.80	28.97	.	28.00
187	**bta-miR-345-5p**	26.88	28.56	27.68	28.02	.	.	27.57	.	27.99	27.86	.	.
188	**bta-miR-369-3p**	33.99	.	.	35.46	31.83	.	35.44	.	35.79	33.69	35.21	34.27
189	**bta-miR-346**	23.23	25.57	23.78	25.34	21.78	23.79	23.98	23.28	24.79	24.82	25.15	24.65
190	**bta-miR-369-5p**	34.30	34.49	33.74	.	32.57	33.90	.	33.91	34.48	34.09	32.95	34.27

191	**bta-miR-34a**	25.49	27.77	25.75	25.68	23.04	25.80	26.19	25.78	26.45	25.91	27.69	25.23
192	**bta-miR-370**	26.11	.	27.00	27.62	25.48	27.24	.	26.70	.	27.83	28.03	26.67
193	**bta-miR-34b**	22.16	24.38	21.71	21.80	20.41	21.37	22.86	22.27	22.85	22.23	24.53	20.84
194	**bta-miR-371**	.	33.27	30.78	31.05	30.62	31.90	30.82	31.09	31.87	32.89	33.03	31.37
195	**bta-miR-34c**	22.15	24.19	21.72	21.69	20.39	21.16	22.98	22.17	22.82	22.12	24.48	20.80
196	**bta-miR-374a**	26.61	28.11	27.15	24.66	24.50	25.42	.	26.60	25.85	25.81	26.32	25.03
197	**bta-miR-361**	26.07	28.01	26.72	25.71	24.62	25.76	26.72	26.20	26.58	26.46	26.86	25.68
198	**bta-miR-374b**	23.74	25.33	24.22	23.74	22.92	23.65	23.59	23.79	24.57	23.72	25.18	23.19
199	**bta-miR-362-3p**	28.96	31.50	30.69	27.79	26.15	28.06	.	29.26	29.29	28.72	29.72	27.74
200	**bta-miR-375**	21.76	22.63	21.64	21.84	21.61	21.83	22.63	21.83	22.79	22.10	23.79	21.68
201	**bta-miR-362-5p**	28.92	31.41	30.27	29.30	27.19	29.10	29.77	29.82	29.88	29.25	30.77	29.29
202	**bta-miR-376a**	33.78	.	34.69	34.96	32.14	.	36.41	.	35.24	34.56	.	34.73
203	**bta-miR-363**	30.30	33.46	31.56	30.32	28.68	29.88	31.50	31.86	30.97	30.30	32.78	29.70
204	**bta-miR-376b**	34.40	34.90	32.94	34.50	33.71	36.08	34.77	35.50	.	36.88	.	33.82
205	**bta-miR-382**	30.33	29.92	29.15	30.81	28.34	28.80	30.60	29.19	30.67	29.73	29.31	28.79
206	**bta-miR-376d**	31.83	34.58	32.69	.	31.84	32.39	32.86	34.17	.	.	.	33.86
207	**bta-miR-383**	.	29.34	28.70	30.13	26.64	29.53	29.18	28.68	29.64	29.75	30.09	.
208	**bta-miR-376e**	33.68	.	33.86	34.13	32.61	35.54	31.32	36.24	.	.	.	32.86
209	**bta-miR-409a**	32.47	34.73	32.97	34.70	.	32.19	33.72	32.08	33.98	.	33.86	32.88
210	**bta-miR-409b**	31.46	34.24	32.81	33.93	29.70	33.98	.	.	32.18	35.27	33.80	32.86
211	**bta-miR-378**	26.59	27.48	26.79	26.46	25.82	25.83	26.70	27.29	27.15	27.40	29.41	25.79
212	**bta-miR-410**	30.75	33.03	.	32.86	.	30.84	30.58	31.61	31.60	32.02	35.36	32.13
213	**bta-miR-378b**	26.81	27.62	27.32	26.45	26.34	25.82	27.52	27.40	27.53	27.42	29.52	25.89
214	**bta-miR-378c**	29.72	31.76	29.68	30.69	.	30.02	30.34	30.58	30.93	30.72	32.91	29.80
215	**bta-miR-411b**	28.86	29.72	29.32	29.61	28.54	29.70	28.95	29.02	29.71	29.55	30.54	29.74
216	**bta-miR-378d**	28.69	29.69	28.79	29.89	26.82	28.71	28.70	29.14	29.73	30.09	.	.
217	**bta-miR-411c-3p**	31.78	35.40	.	33.92	30.35	34.89	.	32.91	33.80	33.89	33.32	32.69
218	**bta-miR-379**	28.26	29.53	28.70	29.67	26.49	29.34	28.02	28.64	28.38	28.73	29.01	29.03
219	**bta-miR-380-3p**	30.74	33.11	31.77	33.14	28.78	31.64	31.76	31.85	33.36	33.26	36.72	.
220	**bta-miR-412**	.	34.11	31.76	35.75	29.71	31.99	32.90	31.84	34.99	.	35.16	32.43
221	**bta-miR-380-5p**	30.00	31.34	30.32	.	28.94	30.30	30.40	30.72	32.67	31.81	30.59	30.51
222	**bta-miR-421**	26.76	28.30	27.10	26.52	25.75	26.59	26.11	26.73	27.31	27.27	28.14	26.58
223	**bta-miR-381**	.	30.86	.	.	28.69	29.75	29.80	31.30	31.31	32.57	31.29	29.92
224	**bta-miR-423-3p**	24.76	25.61	24.83	24.65	23.21	.	24.82	24.72	25.01	24.76	26.05	24.05
225	**bta-miR-423-5p**	23.42	24.48	23.82	24.16	22.82	23.72	23.83	23.73	24.08	23.14	24.83	23.36
226	**bta-miR-449c**	28.11	28.83	28.38	28.29	26.12	27.77	27.79	27.75	28.90	27.92	28.83	26.77
227	**bta-miR-424-3p**	28.90	.	30.75	31.01	27.72	31.94	29.42	30.86	29.71	31.01	32.59	30.79
228	**bta-miR-449d**	25.81	27.73	26.38	27.46	24.39	26.43	26.59	26.63	26.97	27.52	27.59	25.93
229	**bta-miR-424-5p**	24.52	30.14	27.83	26.47	23.40	27.78	25.56	28.50	24.67	26.82	26.73	27.60
230	**bta-miR-450a**	30.71	.	34.50	33.35	30.25	33.98	31.43	35.67	31.78	32.80	32.93	34.79
231	**bta-miR-425-3p**	21.26	22.46	22.10	22.51	20.67	22.78	21.92	21.61	22.32	22.68	22.82	22.21
232	**bta-miR-450b**	32.35	.	.	33.85	31.24	34.55	32.82	34.49	33.65	33.50	33.81	33.57
233	**bta-miR-425-5p**	23.78	24.67	24.57	23.74	22.47	23.56	23.70	23.56	23.81	23.47	24.71	23.49
234	**bta-miR-451**	28.45	30.69	27.91	29.74	24.00	30.07	27.79	28.44	27.57	25.73	29.75	29.68
235	**bta-miR-429**	22.91	24.70	23.89	22.30	21.54	22.57	23.31	22.91	23.34	22.71	23.90	22.14
236	**bta-miR-452**	29.65	31.75	29.59	29.99	27.80	29.71	29.59	29.75	30.00	.	31.56	28.86
237	**bta-miR-431**	28.76	30.34	28.65	.	26.71	28.23	28.69	28.83	30.23	.	30.62	28.71
238	**bta-miR-432**	.	31.05	29.89	.	28.06	30.03	.	.	31.94	30.81	31.59	30.02
239	**bta-miR-433**	27.17	29.75	.	29.71	26.58	28.81	28.22	28.04	.	29.71	.	28.58
240	**bta-miR-454**	32.06	33.41	32.74	33.02	30.53	31.80	31.66	32.82	32.65	31.91	32.83	31.79
241	**bta-miR-448**	32.61	35.73	33.48	34.27	.	33.92	33.74	33.91	33.73	33.96	33.88	32.32
242	**bta-miR-455-3p**	27.40	28.80	27.15	26.83	27.69	27.59	28.50	28.69	28.25	27.55	30.16	27.67
243	**bta-miR-449a**	23.91	25.13	24.31	23.79	21.40	23.62	23.79	22.88	24.76	23.50	24.79	21.94
244	**bta-miR-455-5p**	30.80	33.91	31.83	29.61	28.70	30.25	31.68	31.63	30.20	31.55	32.82	29.60
245	**bta-miR-449b**	26.84	27.63	26.77	26.48	24.31	26.57	26.82	25.80	27.71	26.24	27.42	25.11

246	**bta-miR-483**	29.78	32.25	29.69	32.93	28.45	29.89	30.22	30.50	31.85	32.30	33.67	29.75
247	**bta-miR-484**	26.47	27.51	26.73	27.11	25.13	26.62	.	26.76	27.76	27.15	28.72	26.32
248	**bta-miR-496**	.	33.97	32.14	34.28	30.43	32.92	32.86	34.40	.	.	35.53	32.11
249	**bta-miR-485**	.	36.32	.	36.34	32.05	32.81	33.40	33.92	34.31	35.16	36.30	.
250	**bta-miR-497**	26.52	27.17	26.82	26.79	25.85	26.75	26.70	26.52	27.27	26.71	27.82	26.77
251	**bta-miR-486**	24.09	25.19	24.82	25.14	22.06	24.79	24.73	24.39	24.79	24.95	25.29	24.70
252	**bta-miR-499**	31.81	35.97	33.30	31.90	29.04	30.21	31.74	32.25	32.90	.	32.27	31.52
253	**bta-miR-500**	27.71	29.61	28.25	27.34	25.32	26.93	.	27.57	27.70	27.96	.	27.57
254	**bta-miR-487b**	32.20	36.66	31.89	.	30.78	32.81	32.55	32.54	33.64	33.63	34.13	.
255	**bta-miR-502a**	.	32.02	31.89	32.87	29.59	30.79	32.56	.	31.54	32.01	33.42	32.37
256	**bta-miR-488**	27.79	28.76	28.58	29.03	27.16	28.77	28.39	27.82	28.46	28.69	28.96	28.48
257	**bta-miR-502b**	28.43	.	.	28.16	25.97	27.85	28.74	28.47	28.65	28.18	.	28.06
258	**bta-miR-489**	.	32.80	33.15	32.34	.	33.32	32.70	.	.	33.88	33.99	33.39
259	**bta-miR-503-3p**	25.37	27.25	26.16	26.84	23.32	26.03	26.18	25.72	26.41	26.58	27.26	25.93
260	**bta-miR-490**	27.80	.	28.40	29.10	25.83	28.22	28.62	28.26	29.81	29.22	29.94	28.05
261	**bta-miR-503-5p**	28.79	31.03	29.63	30.41	27.84	30.39	29.81	29.65	30.69	31.33	30.83	30.49
262	**bta-miR-491**	27.47	29.69	27.83	26.37	26.25	26.08	27.64	27.62	27.74	27.64	27.76	25.86
263	**bta-miR-493**	25.21	.	25.52	27.43	22.53	25.65	25.86	25.71	26.80	26.91	26.83	25.23
264	**bta-miR-505**	24.57	25.28	24.99	24.73	23.65	24.52	25.03	24.67	25.84	25.02	25.93	24.66
265	**bta-miR-494**	16.80	17.40	17.99	17.45	12.80	16.78	17.39	16.73	16.54	16.84	16.40	17.25
266	**bta-miR-532**	.	30.04	29.80	29.78	25.95	28.67	29.77	28.81	29.83	29.86	29.26	28.41
267	**bta-miR-495**	34.56	.	32.87	31.99	.	34.00	35.84	34.57	36.89	33.99	.	32.48
268	**bta-miR-541**	27.66	29.18	27.71	28.49	24.41	27.96	27.81	27.81	28.66	28.51	28.54	27.21
269	**bta-miR-584**	.	28.30	27.19	.	25.23	27.37	27.41	.	28.07	28.32	28.49	27.41
270	**bta-miR-543**	33.06	34.97	31.75	.	.	32.77	33.11	32.68	34.05	33.95	.	33.98
271	**bta-miR-592**	29.92	33.56	30.77	28.99	27.74	28.06	31.31	30.30	30.18	.	31.00	29.84
272	**bta-miR-544b**	30.31	34.00	.	35.94	.	30.16	.	31.55	.	35.09	33.88	29.91
273	**bta-miR-615**	11.70	11.79	11.93	11.99	11.81	12.49	11.80	12.00	12.44	12.42	12.52	12.23
274	**bta-miR-545-3p**	32.51	.	36.33	32.92	29.51	32.04	.	33.99	33.41	32.92	.	32.49
275	**bta-miR-628**	29.55	31.75	30.10	29.41	29.21	30.67	29.78	30.40	31.56	30.38	30.71	29.07
276	**bta-miR-545-5p**	34.49	33.99	33.90	33.19	30.42	31.06	35.79	33.85	32.34	34.14	33.87	32.91
277	**bta-miR-631**	19.51	19.67	19.23	19.82	19.79	20.12	19.05	19.46	20.49	19.95	20.16	19.85
278	**bta-miR-551a**	32.93	34.18	31.86	33.94	31.51	32.94	.	33.99	34.18	34.20	36.09	33.06
279	**bta-miR-652**	26.60	27.71	26.91	25.97	24.63	25.76	26.74	25.97	26.77	26.34	27.28	25.74
280	**bta-miR-551b**	33.92	35.70	32.94	32.74	29.94	32.88	32.79	33.89	.	32.50	35.06	.
281	**bta-miR-653**	33.98	36.05	35.49	.	.	.	33.22	36.56	.	.	.	33.87
282	**bta-miR-562**	33.17	.	33.29	.	.	34.93	32.80	35.52	35.95	35.56	33.88	32.09
283	**bta-miR-654**	25.73	27.75	26.14	27.47	25.49	26.72	26.43	26.25	26.86	27.51	28.23	26.23
284	**bta-miR-574**	19.48	20.97	19.76	20.82	18.52	20.12	20.26	19.82	20.76	20.78	21.47	19.79
285	**bta-miR-656**	32.75	33.99	32.92	33.94	30.72	33.73	33.63	32.50	34.18	32.20	33.89	31.84
286	**bta-miR-658**	30.16	.	31.29	31.49	.	30.28	31.09	.	29.98	.	30.93	30.80
287	**bta-miR-758**	.	.	32.76	32.84	31.69	34.43	32.53	.	35.14	33.48	.	32.71
288	**bta-miR-660**	26.01	28.22	26.96	26.14	24.81	26.18	26.11	26.27	26.75	26.17	27.76	26.42
289	**bta-miR-664a**	23.64	24.91	24.54	24.55	22.80	24.31	24.40	24.17	24.42	24.28	24.71	24.51
290	**bta-miR-760-3p**	25.72	27.77	26.66	27.94	24.90	.	26.73	.	27.82	27.77	28.35	26.42
291	**bta-miR-664b**	20.34	21.92	20.78	21.39	19.50	21.40	21.04	20.37	21.10	21.04	21.82	21.33
292	**bta-miR-760-5p**	23.06	24.25	22.75	24.16	21.82	23.22	22.92	22.69	.	23.80	24.84	23.53
293	**bta-miR-761**	29.74	.	30.21	32.00	.	.	30.35	30.18	31.84	31.72	32.11	.
294	**bta-miR-669**	21.71	23.67	22.50	23.32	19.80	22.42	22.71	22.11	22.34	22.86	23.41	22.28
295	**bta-miR-763**	27.39	28.87	27.74	28.80	24.66	27.78	28.02	.	28.51	28.78	.	27.78
296	**bta-miR-670**	.	32.52	34.93	34.81	31.19	31.41	.	32.94	35.53	35.04	32.72	33.85
297	**bta-miR-764**	29.79	30.51	29.79	31.79	.	30.76	29.57	29.86	31.86	30.79	.	31.48
298	**bta-miR-671**	29.43	31.09	29.80	29.76	27.68	28.81	.	29.68	30.46	.	.	29.31
299	**bta-miR-767**	30.44	30.71	30.00	30.47	27.32	30.22	.	30.76	30.08	30.28	30.19	29.84
300	**bta-miR-677**	22.29	23.76	23.75	22.13	20.34	21.20	22.80	22.28	21.83	22.29	22.73	21.67

301	**bta-miR-769**	28.73	30.72	28.72	.	26.71	28.70	29.00	.	29.78	29.74	30.48	28.80
302	**bta-miR-7**	26.88	29.64	27.39	26.52	25.35	25.84	26.86	27.50	27.60	27.42	28.43	25.69
303	**bta-miR-873**	28.93	.	29.81	30.84	28.40	29.77	.	29.79	.	31.57	30.77	.
304	**bta-miR-708**	27.47	28.99	28.42	27.83	25.49	27.35	27.68	27.34	27.73	27.55	27.97	27.72
305	**bta-miR-874**	26.38	27.69	26.84	27.57	24.10	26.60	27.03	26.83	27.72	27.71	28.00	26.76
306	**bta-miR-744**	25.20	26.82	25.66	25.77	24.99	25.73	26.02	25.79	26.24	25.70	26.47	25.38
307	**bta-miR-876**	31.09	35.25	33.11	36.34	30.64	32.85	32.85	.	32.90	35.20	34.40	32.67
308	**bta-miR-98**	26.25	29.10	26.83	26.72	25.00	26.63	26.22	26.68	27.57	26.75	28.50	25.90
309	**bta-miR-877**	23.80	25.35	24.87	24.54	20.73	23.70	24.75	23.80	23.43	24.40	23.99	23.83
310	**bta-miR-99a-3p**	28.15	30.75	28.70	28.62	26.72	28.75	28.65	27.81	29.42	28.55	29.65	28.35
311	**bta-miR-885**	23.64	24.41	23.50	23.95	22.26	24.30	23.70	22.95	24.82	24.09	25.84	24.11
312	**bta-miR-99a-5p**	22.37	23.82	22.78	20.68	19.79	20.75	21.65	21.51	22.07	21.59	22.35	20.79
313	**bta-miR-9-5p**	24.67	28.01	26.17	24.74	23.75	24.59	25.40	25.23	24.89	.	25.20	23.67
314	**bta-miR-1179**	33.43	34.23	32.89	.	.	33.15	33.99	31.84	35.34	.	.	33.63
315	**bta-miR-92a**	21.26	22.38	21.68	21.76	20.49	20.94	21.73	21.37	21.80	21.68	22.78	20.99
316	**bta-miR-92b**	19.90	20.64	19.93	20.61	19.55	20.23	20.68	19.78	20.73	20.35	21.31	19.46
317	**bta-miR-1193**	.	.	34.77	.	31.86	34.24	.	34.98	36.06	35.96	.	.
318	**bta-miR-93**	23.23	24.51	24.06	23.52	21.48	23.27	23.60	23.51	23.45	23.66	23.94	23.27
319	**bta-miR-935**	26.71	.	.	27.78	24.18	27.62	27.45	.	.	27.87	.	27.29
320	**bta-miR-122**	.	35.89	.	34.63	.	33.40	33.84	.	35.64	33.92	.	32.05
321	**bta-miR-940**	21.96	22.75	22.36	22.83	17.78	22.65	22.30	21.87	22.40	22.83	22.70	22.61
322	**bta-miR-1224**	19.99	20.94	21.34	21.07	16.47	20.58	20.71	20.29	19.83	20.20	20.21	20.59
323	**bta-miR-95**	27.20	28.75	26.87	26.20	25.79	25.50	27.57	27.77	28.18	26.63	29.68	25.50
324	**bta-miR-1225-3p**	22.42	23.67	22.78	.	19.84	22.65	22.81	22.73	23.60	23.35	23.80	22.82
325	**bta-miR-96**	28.70	30.21	28.35	26.50	24.55	27.12	27.71	28.46	28.80	28.78	28.75	27.90
326	**bta-miR-1246**	12.64	14.03	13.15	13.49	10.83	13.07	13.25	12.70	12.71	12.91	13.65	13.29
327	**bta-miR-1247-3p**	24.87	26.51	25.74	26.30	22.15	25.81	25.79	25.56	25.74	25.78	25.89	25.83
328	**bta-miR-1296**	24.50	26.89	25.60	26.70	.	25.66	25.89	25.39	25.80	26.29	26.65	25.24
329	**bta-miR-1247-5p**	23.64	24.68	24.69	23.84	19.51	23.78	23.84	23.63	23.65	24.29	23.83	24.17
330	**bta-miR-1298**	30.80	33.94	.	36.62	.	.	.	.	33.85	36.53	32.90	33.44
331	**bta-miR-1248**	19.17	20.51	19.57	20.01	17.19	19.78	19.79	19.39	19.66	19.69	19.90	20.10
332	**bta-miR-1301**	30.85	31.08	31.00	34.03	29.79	30.53	32.18	30.86	32.18	31.85	30.70	29.90
333	**bta-miR-1249**	24.92	25.87	25.60	26.22	24.60	26.12	24.90	24.76	26.37	26.28	26.68	24.83
334	**bta-miR-1306**	25.49	26.77	26.04	26.00	24.76	25.91	26.03	25.73	26.87	26.18	27.61	25.57
335	**bta-miR-1260b**	18.10	17.38	18.80	19.25	16.70	18.72	18.71	18.31	18.69	18.50	18.70	18.69
336	**bta-miR-1307**	19.98	21.05	20.97	23.13	17.78	21.02	20.77	19.96	21.00	21.04	21.50	20.83
337	**bta-miR-1271**	29.81	32.85	29.74	28.88	28.58	28.76	30.69	30.71	30.93	29.96	30.53	28.51
338	**bta-miR-1343-3p**	24.58	25.97	24.81	25.29	23.84	24.69	25.22	24.81	25.62	25.39	26.54	24.49
339	**bta-miR-1343-5p**	23.07	24.03	23.66	23.79	18.83	23.21	23.79	23.37	23.39	23.76	23.42	23.44
340	**bta-miR-1281**	23.35	25.70	23.56	25.30	22.63	24.35	24.11	24.22	25.16	25.61	25.68	23.79
341	**bta-miR-1388-3p**	26.00	27.90	26.66	26.55	23.22	26.63	26.47	26.78	26.67	26.73	27.91	25.81
342	**bta-miR-1282**	31.08	34.49	31.27	33.90	.	32.69	.	31.82	33.58	35.00	34.58	31.81
343	**bta-miR-1284**	29.50	31.00	.	.	30.15	29.45	.	.	31.90	32.84	33.42	29.84
344	**bta-miR-1287**	28.79	31.75	29.77	31.84	29.54	30.42	31.38	30.76	30.79	32.04	32.48	30.18
345	**bta-miR-1291**	21.56	23.34	22.87	22.73	19.24	21.75	22.77	21.73	21.85	22.20	.	22.48
