# Supplementary material for: Changes in Oviductal Cells and Small Extracellular Vesicles miRNAs in Pregnant Cows
Source: Front Vet Sci. 2021 Mar 4;8:639752. doi: 10.3389/fvets.2021.639752 (PMC7969882; doi:10.3389/fvets.2021.639752)
Supplement: Supplementary file 6 [file Table_5.pdf]

S5 Table. Full list of biological pathways with significant p-value ($p<0.05$) predicted as modulated by 6 miRNAs (bta-miR-133b, bta-miR-205, bta-miR-584, bta-miR-551a, bta-miR-1193 and bta-miR-1225-3p) up-regulated in OECs from non-pregnant compared to pregnant cows.

Pathways	Number of genes	P-value
bta04360 Axon guidance	70	0
bta04014 Ras signaling pathway	91	0
bta04010 MAPK signaling pathway	105	0
bta05212 Pancreatic cancer	37	0.0001
bta04062 Chemokine signaling pathway	67	0.0003
bta05220 Chronic myeloid leukemia	34	0.0005
bta04810 Regulation of actin cytoskeleton	71	0.0008
bta04068 FoxO signaling pathway	49	0.0008
bta05214 Glioma	33	0.0009
bta05200 Pathways in cancer	155	0.001
bta04530 Tight junction	61	0.0011
bta05205 Proteoglycans in cancer	68	0.0014
bta00564 Glycerophospholipid metabolism	40	0.0015
bta04722 Neurotrophin signaling pathway	45	0.0016
bta04072 Phospholipase D signaling pathway	53	0.0018
bta04660 T cell receptor signaling pathway	40	0.0023
bta01522 Endocrine resistance	36	0.0027
bta04921 Oxytocin signaling pathway	52	0.0027
bta04012 ErbB signaling pathway	33	0.0028
bta04514 Cell adhesion molecules (CAMs)	53	0.0035
bta04912 GnRH signaling pathway	35	0.0038
bta04928 Parathyroid hormone synthesis. secretion and action	38	0.0039
bta05215 Prostate cancer	36	0.0046
bta04550 Signaling pathways regulating pluripotency of stem cells	48	0.0046
bta04710 Circadian rhythm	16	0.0047
bta04310 Wnt signaling pathway	53	0.0052
bta04611 Platelet activation	42	0.0053
bta05211 Renal cell carcinoma	28	0.0054
bta04925 Aldosterone synthesis and secretion	35	0.0056
bta04020 Calcium signaling pathway	63	0.0061
bta05163 Human cytomegalovirus infection	74	0.0061
bta04934 Cushing syndrome	51	0.0061
bta04071 Sphingolipid signaling pathway	41	0.0072
bta04137 Mitophagy	26	0.0072
bta05225 Hepatocellular carcinoma	55	0.0079
bta04144 Endocytosis	73	0.0082
bta05223 Non-small cell lung cancer	26	0.0083
bta04150 mTOR signaling pathway	50	0.0097
bta04152 AMPK signaling pathway	41	0.0099
bta05224 Breast cancer	48	0.0103
bta04920 Adipocytokine signaling pathway	27	0.0103
bta04261 Adrenergic signaling in cardiomyocytes	48	0.0103
bta04211 Longevity regulating pathway	32	0.0103
bta04024 cAMP signaling pathway	68	0.011
bta04061 Viral protein interaction with cytokine and cytokine receptor	33	0.012
bta05219 Bladder cancer	18	0.0122
bta04919 Thyroid hormone signaling pathway	39	0.0128
bta04668 TNF signaling pathway	39	0.0128

bta05226 Gastric cancer	48	0.0135
bta04926 Relaxin signaling pathway	42	0.0136
bta01100 Metabolic pathways	372	0.014
bta04130 SNARE interactions in vesicular transport	15	0.0143
bta04961 Endocrine and other factor-regulated calcium reabsorption	20	0.0149
bta04390 Hippo signaling pathway	48	0.0176
bta04931 Insulin resistance	36	0.0181
bta04015 Rap1 signaling pathway	63	0.0182
bta05218 Melanoma	26	0.0188
bta04728 Dopaminergic synapse	42	0.0198
bta04520 Adherens junction	25	0.0204
bta05210 Colorectal cancer	30	0.0216
bta04371 Apelin signaling pathway	43	0.0239
bta04659 Th17 cell differentiation	36	0.0244
bta05160 Hepatitis C	49	0.0245
bta04917 Prolactin signaling pathway	28	0.0255
bta04625 C-type lectin receptor signaling pathway	34	0.0261
bta04022 cGMP-PKG signaling pathway	50	0.0265
bta04070 Phosphatidylinositol signaling system	32	0.0279
bta04140 Autophagy	43	0.0282
bta04922 Glucagon signaling pathway	33	0.0283
bta04923 Regulation of lipolysis in adipocytes	21	0.0283
bta00310 Lysine degradation	23	0.0308
bta04911 Insulin secretion	28	0.0317
bta00561 Glycerolipid metabolism	23	0.0348
bta05166 Human T-cell leukemia virus 1 infection	65	0.035
bta04510 Focal adhesion	56	0.0371
bta05202 Transcriptional misregulation in cancer	54	0.0401
bta04933 AGE-RAGE signaling pathway in diabetic complications	32	0.0408
bta05412 Arrhythmogenic right ventricular cardiomyopathy (ARVC)	25	0.041
bta04713 Circadian entrainment	31	0.0444
bta05100 Bacterial invasion of epithelial cells	24	0.0447
bta00562 Inositol phosphate metabolism	24	0.0447
bta04370 VEGF signaling pathway	20	0.0451
bta04910 Insulin signaling pathway	41	0.0454
bta04340 Hedgehog signaling pathway	18	0.0475
bta04350 TGF-beta signaling pathway	29	0.0479
bta04670 Leukocyte transendothelial migration	34	0.0491
