# Supplementary figures and images for: Changes in Oviductal Cells and Small Extracellular Vesicles miRNAs in Pregnant Cows
Source: Front Vet Sci. 2021 Mar 4;8:639752. doi: 10.3389/fvets.2021.639752 (PMC7969882; doi:10.3389/fvets.2021.639752)

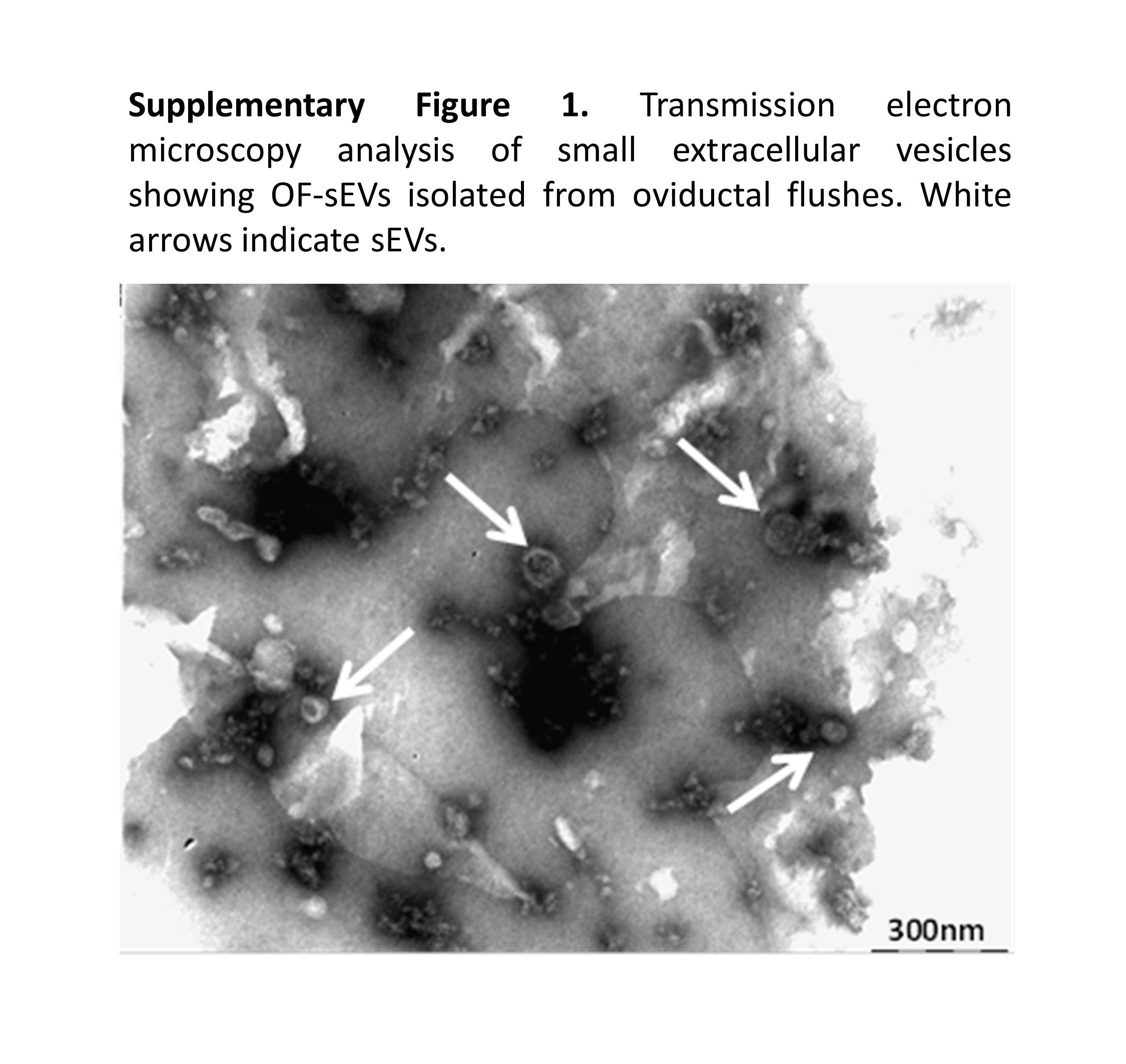

Supplement: Supplementary file 7 [file Image_1.tif]
